# Cyclic Expression of Lhx2 Regulates Hair Formation

**DOI:** 10.1371/journal.pgen.1000904

**Published:** 2010-04-08

**Authors:** Gunilla Törnqvist, Anna Sandberg, Anna-Carin Hägglund, Leif Carlsson

**Affiliations:** Umeå Center for Molecular Medicine, Umeå University, Umeå, Sweden; Stanford University School of Medicine, United States of America

## Abstract

Hair is important for thermoregulation, physical protection, sensory activity, seasonal camouflage, and social interactions. Hair is generated in hair follicles (HFs) and, following morphogenesis, HFs undergo cyclic phases of active growth (anagen), regression (catagen), and inactivity (telogen) throughout life. The transcriptional regulation of this process is not well understood. We show that the transcription factor Lhx2 is expressed in cells of the outer root sheath and a subpopulation of matrix cells during both morphogenesis and anagen. As the HFs enter telogen, expression becomes undetectable and reappears prior to initiation of anagen in the secondary hair germ. In contrast to previously published results, we find that Lhx2 is primarily expressed by precursor cells outside of the bulge region where the HF stem cells are located. This developmental, stage- and cell-specific expression suggests that Lhx2 regulates the generation and regeneration of hair. In support of this hypothesis, we show that Lhx2 is required for anagen progression and HF morphogenesis. Moreover, transgenic expression of Lhx2 in postnatal HFs is sufficient to induce anagen. Thus, our results reveal an alternative interpretation of Lhx2 function in HFs compared to previously published results, since Lhx2 is periodically expressed, primarily in precursor cells distinct from those in the bulge region, and is an essential positive regulator of hair formation.

## Author Summary

Hair is generated in hair follicles, complex mini-organs in the skin that are devoted to this task. All hair follicles are generated during embryonic development. The hair follicles generate a new hair shaft by cycling through stages of regression, rest, and growth continuously throughout life. The length of the growth phase determines the length of the hair. The reason(s) for this complicated regulation of hair growth is not clear, but it has been suggested that it may accommodate seasonal variations in hair growth. In this study we have identified the transcription factor Lhx2 as an important regulator of hair formation. The *Lhx2* gene is active during the growth phase of the hair follicle and is turned off during the resting phase. We confirm that Lhx2 is functionally involved in hair formation, since hair follicles where Lhx2 has been inactivated are unable to make hair. Moreover, activation of the Lhx2 gene in hair follicles induced the growth phase and hence hair formation. Thus, Lhx2 is an important regulator of hair growth.

## Introduction

Hair develops in hair follicles (HFs) and embryonic development of HFs (morphogenesis) is initiated between embryonic days 12 (E12) to E15, and is regulated by continuous epithelial-mesenchymal interactions between epidermal and dermal cells [1]–[3]. The first morphological sign of HF morphogenesis is a thickening of the epithelial cell layer leading to the formation of a placode. The placode induces a dermal condensation in the underlying mesenchyme that signals to placodal cells to proliferate and grow down into the dermis forming the primary hair germ (HG), followed by the formation of the hair peg. Subsequently, at the bulbous peg stage, the dermal condensate forms the dermal papilla (DP) as it becomes enveloped by follicular epithelial cells [2]. At this stage the proliferating and differentiating epidermal matrix cells adjacent to the DP start to generate the different layers of the HF consisting of the medulla, cortex and cuticle of the hair shaft, and the cuticle, Huxley's layer and Henle's layer of the inner root sheath (IRS). The IRS is surrounded by a distinct outer layer of outer root sheath (ORS) cells consisting of a layer of epidermal cells continuous with the epidermis [3],[4].

After birth, HFs cycle through stages of active growth (anagen), regression (catagen) and inactivity (telogen) [2]. The periodic growth and rest of the HF reflects the migration, proliferation and differentiation of multipotent HF stem cells suggested to be located primarily in the bulge region of the ORS [5]. Multipotent stem cells have also been detected outside of bulge region in the lower part of whisker HFs [6], and the matrix in pelage HFs is suggested to contain restricted self-renewing stem cell for each inner structure [7]. Whether these observations reflect differences in stem cell migration and differentiation between whisker and pelage HF remains to be elucidated. The onset of anagen is characterised by the initiation of cell proliferation in the secondary HG adjacent to the DP in the proximal part of the HF [8], leading to the invasion of the elongating HF into subcutaneous tissue. This process is accompanied by the differentiation of the matrix cells in the hair bulb leading to the formation of the hair shaft and the IRS. In pigmented animals melanins are synthesised by melanocytes located close to the forming hair shaft and this process occurs exclusively during anagen [9]. During the following catagen phase, hair shaft production stops since proliferation and differentiation is dramatically reduced leading to a regression of the HF. Catagen is followed by telogen which is characterised by a minimal signalling exchange between the DP and follicular keratinocytes [10].

Numerous signalling pathways have been implicated in the regulation of the HF and considerable overlap in the pathways promoting both HF morphogenesis and the anagen stage of postnatal HF cycling has been revealed [3]. Signalling induced by Sonic hedgehog (Shh) and Wnts are indispensable for HF morphogenesis and the anagen phase of HF cycle [11]–[14]. Active Wnt signalling leads to stabilization and nuclear translocation of cytoplasmic β-catenin that activate transcription of specific target genes together with the Lef1/Tcf proteins [15]. Activation of hedgehog signalling occurs upon ligand-binding to Patched (Ptc) that subsequently releases its inhibitory effect on Smoothened (Smo) leading to transcription of specific target genes by the Gli family of transcription factors [16]. Inhibition of Bone morphogenetic protein (BMP) signalling by Noggin (Nog) is important for both morphogenesis and anagen induction [17],[18].

Mediators of paracrine and autocrine interactions trigger intracellular signals that are transmitted to the nucleus, where the activation of specific transcription factors establish and propagate proper epithelial-mesenchymal interactions during organ generation and regeneration. Therefore, it is important to identify and characterize the transcription factors that are critically involved in the development of HF as well as the regulation of HF cycling to further elucidate epithelial-mesenchymal interactions specific for HF morphogenesis and cycling. One class of transcription factors, the LIM-homeodomain family, regulates many important developmental processes such as asymmetric cell division, tissue specification and differentiation of specific cell types [19]. One member of this gene family, *Lhx2* (previously *LH2*, *LH2A*), was first identified as a gene specifically expressed in pre-B cell lines and independently isolated as a transcription factor binding to the glycoprotein hormone α-subunit promoter [20],[21]. Lhx2 has been shown to be essential in various epithelial-mesenchymal interactions as well as in the regulation of different progenitor/stem cell populations [20], [22]–[27], revealing its importance in regulating fundamental processes important for organ/tissue generation and regeneration.

Since Lhx2 plays an important role in a variety of epithelial-mesenchymal interactions and in the regulation of various stem/progenitor cell populations, we analysed the expression pattern and function of Lhx2 in the HF. Based on mRNA expression, Lhx2 is expressed from an early stage of morphogenesis and eventually becomes restricted to the ORS and a subpopulation of the matrix cells located in the proximal part of the hair bulb. During postnatal HF cycling Lhx2 expression reveals a similar pattern and presence of Lhx2 protein in matrix cells and in cells scattered in the ORS was confirmed. Moreover, Lhx2 expression is associated with the anagen phase and was also primarily expressed by precursor cells outside of the bulge region. These results indicate that Lhx2 is involved in anagen initiation/progression and morphogenesis and contradict previously published data where Lhx2 protein was only detected in stem cells in the bulge region [27]. Rhee et al. also suggested that Lhx2 was important for maintaining quiescent stem cells and not involved in their differentiation. However, by using mouse models where Lhx2 expression could be conditionally inactivated in postnatal HFs, or significantly reduced during morphogenesis, we could confirm that Lhx2 is important for both anagen and morphogenesis progression. Furthermore, by using a mouse model where Lhx2 expression could be induced in postnatal HFs, we also showed that Lhx2 expression was sufficient to induce anagen. Thus, cyclic expression of Lhx2 in HFs regulates hair formation.

## Results

### Expression of Lhx2 during HF morphogenesis and postnatal cycling

**Figure 1:**
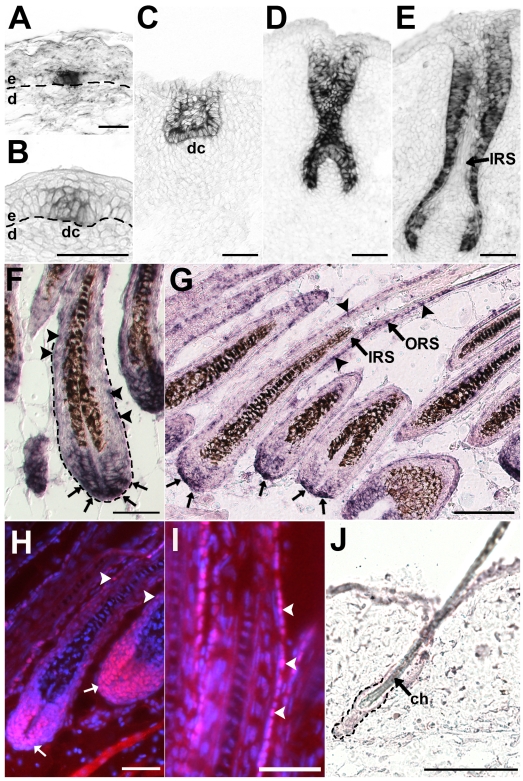
Lhx2 is expressed from early stages of morphogenesis and becomes restricted to the proximal part of the hair bulb and the ORS in fully developed HFs. Lhx2 expression analysed by in situ hybridization (A–G,J) and immunohistochemistry (H-I) of tissue sections of HFs at different stages of morphogenesis (A–F) and in postnatal anagen (G–I) and telogen (J). (A) Lhx2 expression in basal keratinocytes at the pre-germ stage (Stage 0) prior to any obvious morphological change of the keratinocytes or the underlying dermis. (B) HF at the hair germ or placode stage (Stage 1) showing Lhx2 expression in the keratinocytes located at the local thickening of the epidermis. Reorganisation of mesenchymal cells in the dermis beneath the placode is indicative of formation of a dermal condensate. (C) HF at germ/peg stage (Stage 2–3) of HF morphogenesis. (D) HF at the peg stage (Stage 4), Lhx2 is expressed in the entire epithelial portion of the HF. (E) HF at the bulbous peg stage (Stage 5–6) when formation of the IRS has begun. Lhx2 is still widely expressed in outer layers of the HF and in the proximal extension the ORS in the future hair bulb whereas expression is turned off in cells in the forming IRS. (F) Fully developed HFs when the hair shaft has erupted through the epidermis (Stage 8). Lhx2 is expressed in the proximal part of the hair bulb (arrows) and in cells scattered in the ORS (arrow heads). (G) Analysis of Lhx2 expression in HFs during postnatal anagen Sub-stage VI revealing expression in the proximal part of the hair bulb (arrows) and in cells scattered in the ORS (arrow heads). (H,I) Immunohistochemical analysis of Lhx2 expression in anagen Sub-stage VI HFs revealing presence of nuclear Lhx2 protein in cells in proximal part of the hair bulb and in matrix cells (H, arrows) and in cells scattered in the ORS (H and I, arrow heads). (J) Analysis of Lhx2 expression in adult HFs in the extended telogen in 7–8 weeks old mice revealing no detectable expression. ch, club hair; dc. dermal condensate; d, dermis; e, epidermis. Scale bars, (A–I) 50 µm; (J) 100 µm.

To elucidate the expression pattern of *Lhx2* in detail we used in situ hybridization analysis of HFs during different stages of morphogenesis. *Lhx2* starts to be expressed in patches of basal epidermal cells prior to any obvious formation of a dermal condensate in the underlying dermis (Stage 0 of HF morphogenesis, see [28]) (Figure 1A). *Lhx2* is subsequently expressed in the placode when the epidermal thickening and the adjacent dermal condensate have formed (Stage 1) (Figure 1B), and in the downward growing primary HG (Stage 2–3) (Figure 1C). This expression pattern of Lhx2 mRNA in the developing HF is in agreement with what has been reported previously [27]. At the epithelial peg stage (Stage 4) *Lhx2* is expressed by most cells in the epithelial portion of the developing HF (Figure 1D), and we have no evidence for higher expression at the leading edge of the HF as previously reported [27]. At the stage when the concentric layers of the HF are initially formed (bulbous peg, Stage 5), *Lhx2* expression becomes down-regulated in the differentiated cells in the IRS (Figure 1E). During the postnatal phase of morphogenesis when the HFs are fully developed and the hair shaft has erupted through the skin surface (Stage 8), *Lhx2* expression is maintained predominantly in the epidermal matrix cells located at the most proximal portion of the hair bulb and *Lhx2^+^* cells are also scattered within the ORS (Figure 1F). A similar expression pattern is also seen during whisker follicle morphogenesis (Figure S1A). This expression pattern of Lhx2 has not been described previously and we did not observe an enrichment of *Lhx2* expressing cells in the upper ORS at the presumptive bulge region [27].

In situ hybridization analysis of postnatal HFs in full anagen (Sub-stage VI, [29]) showed that *Lhx2* expression was primarily detected in matrix cells in the proximal part of the bulb region and in cells scattered in the ORS (Figure 1G). This expression pattern is almost identical to that during late morphogenesis (Figure 1F), and is also similar to the expression pattern in adult whisker follicles which are characterised by an extended anagen (Figure S1B) [30]. We also detected Lhx2 protein in the nucleus of cells located in the proximal part of the hair bulb and in cells scattered in the ORS in anagen HFs (Figure 1H and 1I). The Lhx2 protein appears to be more stable compared to Lhx2 mRNA since the protein can also be detected in matrix cells as they differentiate and move distally in the hair bulb, whereas the mRNA is enriched in the cells from where the matrix cells originate in the proximal part of the bulb (Figure 1H and 1G). Persistence of Lhx2 protein after mRNA down-regulation during differentiation has been observed previously in the olfactory epithelium [24]. Thus, the presence of Lhx2 mRNA in the HF is a reliable indicator of functional expression of Lhx2. However, *Lhx2* expression was not detected at the time when the HFs had entered telogen at 7–8 weeks of age, (Figure 1J), suggesting a cyclic expression pattern of *Lhx2* in postnatal HFs.

**Figure 2:**
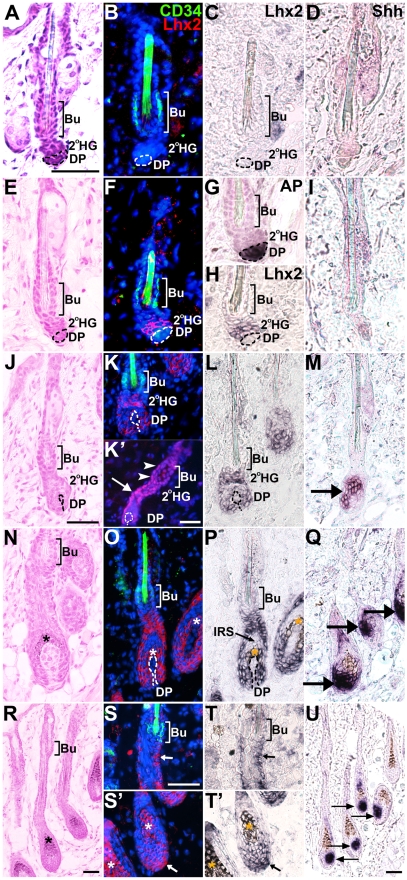
Lhx2 expression is associated with anagen during postnatal HF cycling and is not transcribed by CD34^+^ bulge cells. HFs at different Stages and Sub-stages of postnatal cycles analysed by Hematoxylin/Eosin (H/E) staining (A,E,J,N,R), CD34 immunofluorescence (B,F,K,O,S,S’), Lhx2 in situ hybridisation (B,C,F,H,K,L,O,P,S,S’,T,T’), Lhx2 immunofluorenscence (K’), Shh in situ hybridisation (D,I,M,Q,U) and alkaline phosphatase (AP) staining (G). The CD34 immunoflouresence and Lhx2 in situ hybridisation are performed on the same section and the Lhx2 in situ signal (black) has been pseudocoloured red on the DAPI stained sections (B,F,K,O,S,S’). AP staining distinguishes the DP from the secondary HG where Lhx2 is initially expressed in late telogen (F–H). Shh expression confirms the stage of the HF cycle since Shh is only expressed during anagen. (A–D) HFs in early telogen, CD34^+^ cells are located in the bulge (Bu) area (B) and no Lhx2 expression (B and C) or Shh expression (D) can be detected at this stage. (E–I) HFs in late telogen, CD34^+^ cells are located in the bulge area (F) whereas Lhx2 is expressed by cells in the secondary hair germ (2°HG) located between the bulge area and the AP^+^ cells in the DP (F–H). There is no overlap in Lhx2 and CD34 expression (F). Shh is not expressed confirming that the HFs are in telogen (I). (J–M) HFs in anagen Sub-stages I-II as no deposition of pigment is detected (J). CD34^+^ cells are located in the bulge area whereas Lhx2 is expressed in the secondary HG and the extended part of the HF enveloping the DP (K and L). Immunohistochemical analysis of Lhx2 protein reveal presence of Lhx2 in the 2°HG (K’, arrows) as well as a few cells in the lower part of the bulge region (K’, arrow heads), despite absence of Lhx2 mRNA in this part of the HF. Shh is expressed confirming that anagen has commenced (M, arrow). (N–Q) HFs in anagen Sub-stage III as deposition of pigment has started (N). CD34^+^ cells are located in the bulge area (O) whereas Lhx2 expression is detected in the lower transient part of the HF (O,P). Shh is expressed confirming ongoing anagen (Q, arrows). (R–U) HFs in anagen Sub-stage IV-V as the hair shaft has reached the hair canal (R). CD34^+^ cells are located in the bulge area whereas Lhx2 expression is detected in the proximal part of the hair bulb (S’ and T’, arrow) and in cells scattered in the ORS (S and T, arrow). Shh is expressed confirming ongoing anagen (U, arrows). * indicates melanin deposition. Scale bars, 50 µm. 2°HG, secondary hair germ; Bu, bulge; DP, dermal papilla; IRS, inner root sheath.

To further investigate the fluctuations in Lhx2 expression we determined the expression pattern around the time of telogen-anagen transition. Since induction of the second postnatal anagen was difficult to predict in our control animals (Table S1), we analysed HFs during the first postnatal telogen and the different Sub-stages of the subsequent and first postnatal anagen in 3–4 week old mice. During telogen in 3 week and 1 day old mice (Figure 2A and 2D; Table S1), *Lhx2* expression could not be detected in any HF (Figure 2B and 2C; Figure S3A), similar to what was observed in HFs in telogen in 7–8 weeks old mice (Figure 1J). In slightly older animals (≥3 weeks and 4 days) HFs in several individuals have initiated anagen (Table S1). However, some animals at this age that neither show any obvious morphological transition to anagen, nor express anagen-specific genes such as *Shh* (Figure 2E and 2I), revealed distinct expression of *Lhx2* in the secondary HG adjacent to the DP (Figure 2F–2H; Figure S3B). HF stem cells located in the bulge area express CD34, whereas the cells in the secondary HG are CD34^−^ (Figure 2B and 2F) [31]. Few, if any, of the *Lhx2* expressing cells are CD34^+^ since they appear as separate cell populations (Figure 2F). In the early stages of anagen when *Shh* is expressed but prior to any pigment deposition (e.g. anagen Sub-stages I-II. Figure 2J and 2M), *Lhx2* is expressed both by cells in the secondary HG, where the first proliferating cells are located during anagen induction [8], and by the future matrix cells surrounding the DP (Figure 2K and 2L; Figure S3C). Although there is a sharp boundary where Lhx2 mRNA is present or absent between the secondary HG and the bulge region, we can detect Lhx2 protein in a few cells in the lower part of the bulge region as well as in the secondary HG (Figure 2K’). In the subsequent Sub-stage of anagen (maintained *Shh* expression) when melanocytes deposit pigment (Sub-stage IIIa-c, Figure 2N and 2Q), the matrix cells in the proximal part of the bulb and cells in the ORS express *Lhx2* whereas expression is turned off in the IRS cells (Figure 2O and 2P; Figure S3D), similar to the expression pattern during early HF morphogenesis (compare Figure 2P and Figure S3D to Figure 1D and 1E). At later Sub-stages of anagen (Sub-stages IV-VI with maintained *Shh* expression, Figure 2R and 2U) *Lhx2* expression is maintained in the ORS (Figure 2S and 2T; Figure 1G and 1H) and in matrix cells in the most proximal part of the bulb (Figure 2S’ and 2T’; Figure 1G–1I), similar to expression pattern during Stage 8 of HF morphogenesis (Figure 1F). Also during anagen most of the Lhx2^+^ cells are CD34^−^ as they represent separate cell populations (Figure 2K, 2O, 2S, and 2S’). Thus, during postnatal HF cycling, *Lhx2* expression is initiated in a distinct subpopulation of CD34^−^ epidermal progenitor cells in the secondary HG during late telogen immediately prior to anagen induction and the expression is maintained in the transient portion of the HF throughout anagen. In contrast to previously published results [27], the anagen-associated expression during adult HF cycling, and the similarity of this expression pattern to that during HF morphogenesis, suggest that Lhx2 regulate hair generation and regeneration.

### *Lhx2* is required for postnatal hair formation

**Figure 3:**
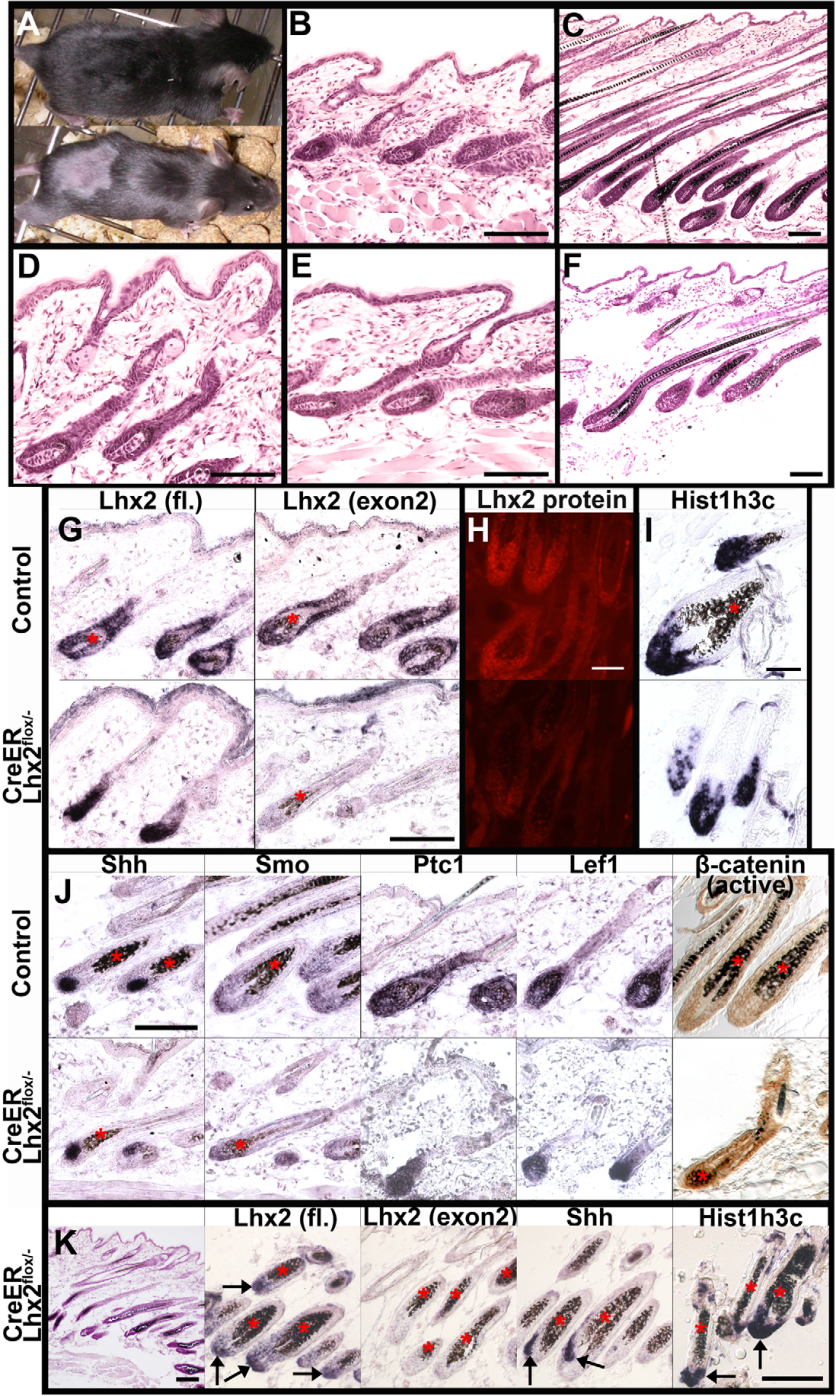
Lhx2 is required for anagen progression. (A) A *Lhx2^flox/-^* control mouse (upper panel) and a *CreER:Lhx2^flox/-^* mouse (lower panel) that were shaved and treated with Tx during the first postnatal telogen and analysed during late first postnatal anagen. Hair have re-grown on the shaved area in the control mouse but not in the *CreER:Lhx2^flox/-^* mouse. (B,C) H/E staining of sections of skin from Tx-treated control mice in early anagen (B, Sub-stage III) and late anagen (C, Sub-stage VI). (D–F) H/E staining of sections of skin from Tx-treated *CreER:Lhx2^flox/-^* mice within the Tx-treated area (D,E) and outside of the Tx-treated area (F). Mutant HFs initiated anagen and developed to Sub-stage III similar to control mice (D, early anagen), but these HFs were arrested at this stage and were unable to assemble a normal hair shaft (E, late anagen). Hair shafts developed in HFs outside of the Tx-treated area in the *CreER:Lhx2^flox/-^* mice (F). (G) In situ hybridization analyses of Lhx2 expression in HFs in anagen in Tx-treated control animals (upper panels) and *CreER:Lhx2^flox/-^* animals (lower panels) using a full length (fl) Lhx2 cDNA probe (left panels) or a probe restricted to exon 2 (right panels). Control HFs revealed hybridization to both probes whereas the mutated HFs only hybridized to the full length probe confirming the expression of the truncated Lhx2 mRNA in the HFs where *Lhx2* has been conditionally inactivated. (H) Immunohistochemical analysis of Lhx2 expression using and anti-Lhx2 antibody in control HFs (upper panel) and in HFs where Lhx2 has been conditionally inactivated (lower panel). (I) In situ hybridization analysis of expression of the S-phase-specific gene *His1h3c* in control anagen HFs (upper panel) and in anagen HFs where Lhx2 has been conditionally inactivated (lower panel). (J) Comparison of gene expression using in situ hybridization of the indicated genes between control anagen HFs (upper panel) and anagen HFs where Lhx2 has been conditionally inactivated (lower panel). Comparison of accumulation of the activated form of β-catenin (brown staining) in control HFs (upper right panel) and in HFs where Lhx2 has been inactivated (lower right panel) using an antibody specific for the dephosphorylated form of β-catenin. (K) Morphological and gene expression analyses of a Tx-treated *CreER:Lhx2^flox/-^* mouse that have not re-grown the hair at 9 week of age. H/E staining of sections of skin revealing that normal hair shafts are not assembled (left panel, compare to control skin in C). Cells in the mutant HFs express Lhx2 mRNA that hybridize to the full length Lhx2 probe (fl. arrows) but not the exon-2 specific probe revealing that the Lhx2 gene is completely inactivated. Cells in mutant HFs also express the anagen-specific gene *Shh* (arrows) and the S-phase-specific gene *His1h3c* revealing the presence of proliferating cells (arrows). * indicates melanin deposition. Scale bars 100 µm.

We have shown that *Lhx2* expression is normally associated with the anagen phase of postnatal HF cycling and we therefore wanted to elucidate whether *Lhx2* is necessary for anagen initiation and/or progression. To address this issue we obtained two mouse strains in which Lhx2 exons have been flanked by *loxp* sites, e.g. floxed *Lhx2* alleles (*Lhx2^flox^*) (Figure S4B, and [32]), in order to conditionally inactivate the *Lhx2* gene by expression of the Cre recombinase [32]. The *Lhx2^flox^* mice were bred into mice harbouring the null allele of *Lhx2* (*Lhx2^−^*) [22], and into transgenic mice ubiquitously expressing a fusion protein between the Cre recombinase and the ligand binding domain of the Estrogen Receptor (*CreER*) [33], to generate *CreER:Lhx2^flox/flox^*, *CreER:Lhx2^flox/-^*, *Lhx2^flox/flox^* and *Lhx2^flox/-^* mice. Application of Tamoxifen (Tx) to the skin leads to nuclear translocation of the CreER fusion protein and conditional inactivation of the *Lhx2* gene in HFs in the *CreER:Lhx2^flox/flox^* and the *CreER:Lhx2^flox/-^* mice. The *Lhx2* gene in the *Lhx2^flox/flox^* and the *Lhx2^flox/-^* mice is unaffected by this treatment and were used as a control. The skin on the back of these mice was shaved and treated with Tx during the first postnatal telogen at approximately 3 weeks of age (Figure S2). Initiation and progression of the first postnatal anagen starting around 4 weeks of age was subsequently analysed (see Figure S2). Most of the Tx-treated *CreER:Lhx2^flox/-^* and *CreER:Lhx2^flox/flox^* mice did not re-grow their hair coat on the shaved area (6/8) whereas all the control animals did (9/9) (Figure 3A), revealing that *Lhx2* is required for postnatal hair regeneration. Histological analysis of the HFs where *Lhx2* was inactivated showed that anagen is initiated similar to control animals and progress to anagen Sub-stage III (Figure 3B and 3D), but whereas the control HFs develop normally the mutant HFs are unable to develop beyond Sub-stage III and assemble a normal hair shaft (Figure 3C and 3E). HFs outside of the Tx-treated area in these mice assembled hair shafts that were indistinguishable from control HFs (Figure 3F). To confirm the inactivation of the *Lhx2* gene we performed in situ hybridisation using the full length *Lhx2* cDNA as a probe which has been shown to hybridise to mRNA expressed from both the WT allele as well as to the inactivated Lhx2 allele [34],[35], and an exon 2-specific probe that only hybridises to the WT mRNA as this exon is deleted in both the conditionally inactivated *Lhx2^flox^* alleles and in the *Lhx2^-^* allele (Figure S4B, [22],[32]). As expected, cells in control HFs in anagen expressed mRNA hybridising to both probes (Figure 3G, left panels), whereas cells in anagen HFs, in which Lhx2 should be conditionally inactivated, expressed a truncated mRNA hybridising exclusively to the full length probe and hence lacking exon 2 (Figure 3G, right panels). Significant reduction of Lhx2 protein in the ORS and the hair bulb could also be observed in the HFs where Lhx2 had been conditionally inactivated (Figure 3H), confirming that the cells expressing the truncated mRNA are unable to generate a functional protein. These results confirmed that the *Lhx2* gene has been inactivated in the Tx-treated *CreER:Lhx2^flox/-^* and *CreER:Lhx2^flox/flox^* mice. Two Tx-treated *CreER:Lhx2^flox/flox^* mice started to re-grow some hair one week later compared to the control animals. Also skin from these individuals had HFs with arrested development, but there were numerous HFs containing cells expressing the control allele since the mRNA hybridised to both the full length probe as well as the exon 2-specific probe (Figure S5). None of the Tx- treated *CreER:Lhx2^flox/-^* mice re-grew their hair, revealing that conditional inactivation of the *Lhx2* gene was more efficient in these mice compared to the *CreER:Lhx2^flox/flox^* mice, where a few cells retaining the control allele could rescue hair formation to some extent. Thus, *Lhx2* is critically involved in hair formation by regulating anagen progression.

The phenotype in *Lhx2^−/−^* mouse forebrain has been suggested to be due to lack of proliferating progenitor cells [22]. To analyse if the HF phenotype is solely due to lack of proliferating progenitor cells, we analysed the expression of the S-phase-specific histone gene *Hist1h3c* [36],[37]. As expected *Hist1h3c* expression could not be detected in HFs in telogen whereas it is expressed in the proximal part of the hair bulb and in matrix cells in HFs in anagen (Figure S6A and S6B; Figure 3I), where the proliferating progenitor cells are located. Numerous cells expressing *Hist1h3c* could also be observed in a similar pattern in the HFs where *Lhx2* had been conditionally inactivated 1–2 weeks after the control mice re-grew their hair (Figure 3I; Figure S6C). Thus, although we have not quantified the level of expression of *Hist1h3c* or quantified the number of proliferating cells, the developmental arrest at anagen Sub-stage III in mutant HFs cannot solely due to the fact that the progenitor cells are unable to proliferate.

The presence of a significant number of proliferating progenitor cells in mutant HFs suggested that the signalling pathways critical for anagen initiation/progression were relatively unperturbed. To address this issue we analysed the expression of various genes encoding mediators of such pathways. No obvious differences in the expression of mediators of hedgehog signalling was observed since the ligand *Shh*, the signal transducer *Smo*, and the receptor and universal target of hedgehog signalling *Ptc1* were equally expressed in control and mutated HFs (Figure 3J). Moreover, the basal components of the canonical Wnt signalling pathway were also present, since both control and mutated HFs accumulated activated (i. e. dephosphorylated) β-catenin, and expressed Lef1, the transcriptional effecter of activated β-catenin (Figure 3J). Thus, inactivation of *Lhx2* appears not to have a major impact on the hedgehog or Wnt signalling pathways.

Since the mutated HFs contained proliferating cells and had no obvious defects in signalling pathways important for anagen progression, we wanted to elucidate if the mutated HFs are either completely blocked or delayed in their development. To address this issue we analysed mice with mutated HFs that had not re-grown any hair at 9 weeks of age. At 9 weeks of age most control animals have entered telogen (Table S1) and thus do not express *Lhx2*, *Shh*, or *Hist1h3c* (Figure 1J; Figure 2B and 2C; Figure 2D and 2I; Figure S6A). Although many HFs have initiated hair shaft formation in the 9 week old conditional mutants, we rarely find any HFs that have developed beyond anagen Sub-stage III and some of the formed hair shafts appear distorted (Figure 3K). Moreover, all HFs contain cells expressing the truncated Lhx2 allele since the mRNA hybridised only to the full length probe whereas it does not hybridise to the exon 2-specific probe (Figure 3K). Also the anagen-specific marker Shh and the proliferation-specific marker Hist1h3c are expressed in the mutated HFs (Figure 3K). These results suggest that the HFs where *Lhx2* were efficiently inactivated are maintained in an anagen-like stage but are unable to assemble a normal full length hair shaft even after an extended period of time. Thus, Lhx2 does not primarily regulate the proliferation of HF progenitor cells but rather appears to regulate the patterning/differentiation of HF progenitor cells.

### Lhx2 is required for HF morphogenesis

**Figure 4:**
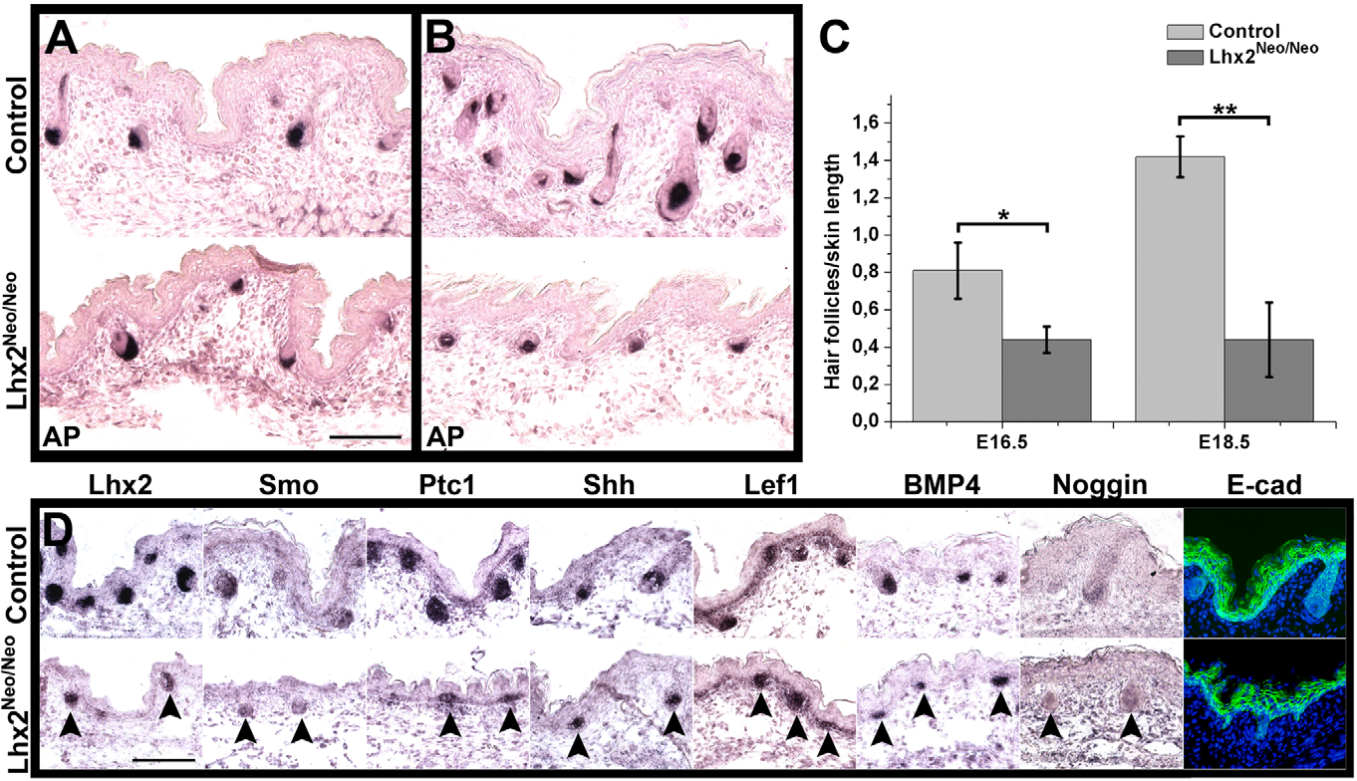
Reduced expression of Lhx2 hampers HF morphogenesis. (A,B) AP staining of skin sections of E16.5 embryos (A) and E18.5 embryos (B) revealing the number of HFs in skin of control embryos (upper panels) compared to skin in *Lhx2^Neo/Neo^* embryos (lower panels). (C) Estimation of HF density based on the AP staining of skin of E16.5 embryos (left) and E18.5 embryos (right) comparing control embryos to *Lhx2^Neo/Neo^* embryos. Data are presented as average ± SD. *p<0.005. **p<0.0001 (Student’s t-test). (D) Skin sections of E16.5 embryos were analysed by in situ hybridization for expression of the indicated genes encoding mediators of signaling pathways involved in HF morphogenesis (arrow heads), and analysed by immunohistochemistry for expression of E-cad in epidermis comparing control embryos (upper panels) to *Lhx2^Neo/Neo^* embryos (lower panels). Scale bars, 100 µm.

Since there is significant overlap between the signalling pathways promoting HF morphogenesis and the anagen stage [3], we wanted to elucidate if Lhx2 is also important for HF morphogenesis. A significant decrease in the number of HFs in *Lhx2^−/−^* embryos has been reported [27], indicating that *Lhx2* play a role in HF morphogenesis. However, since the lethality of *Lhx2^−/−^* embryos at E15–17 [22] coincides with and even precedes the onset of pelage HF morphogenesis, it is difficult to ascertain the precise role of Lhx2 in HF morphogenesis in *Lhx2^−/−^* embryos. To further investigate this we generated a mouse strain harbouring a hypomorphic allele of *Lhx2* denoted *Lhx2^Neo^* (Figure S4A). Mouse embryos homozygous for this allele (*Lhx2^Neo/Neo^* mice) develop an eyeless phenotype similar to the *Lhx2^−/−^* mouse embryos (Figure S4C) confirming that Lhx2 expression was significantly reduced in the *Lhx2^Neo/Neo^* embryos. However, *Lhx2* expression is detected in the *Lhx2^Neo/Neo^* embryos revealing that it is not a null allele (Figure 4D). Since the liver was less affected in these animals compared to *Lhx2^−/−^* embryos (data not shown), the expected number of viable *Lhx2^Neo/Neo^* embryos were obtained at late gestation. Thus, this mouse strain allowed us to follow pelage HF morphogenesis up to a stage when it is well established [2],[38]. Back skin of all E16.5 *Lhx2^Neo/Neo^* embryos contained fewer HFs compared to control animals (Figure 4A and 4C), which is in agreement with the number found in *Lhx2^−/−^* embryos at a similar gestational age [27]. Moreover, the HFs that did develop in the *Lhx2^Neo/Neo^* embryos had a distinct DP but appeared to be developmentally arrested prior to the epithelial peg stage (Stage 4) compared to the HFs in control animals (Figure 4A). At E18.5 the difference in number of HFs between control and *Lhx2^Neo/Neo^* embryos was more striking since HF density did not increase between E16.5 and E18.5 in *Lhx2^Neo/Neo^* embryos (Figure 4B and 4C), whereas it increased by 75% in the control embryos (Figure 4C). Moreover, many HFs in E18.5 *Lhx2^Neo/Neo^* embryos appeared to be developmentally arrested at the same stage as those in the E16.5 embryos (Figure 4A and 4B), similarly to the arrested anagen progression when *Lhx2* is conditionally inactivated in postnatal HFs (Figure 3E). To investigate whether loss of Lhx2 affected signalling pathways critically involved in HF morphogenesis, we analysed expression of various genes encoding mediators of such pathways. Similar to the postnatal HFs where Lhx2 had been conditionally inactivated, there was no obvious difference between control and *Lhx2^Neo/Neo^* HFs in expression levels in for components of the hedgehog signalling pathways (Smo, Shh and Ptc1), the canonical Wnt signalling pathway (Lef1) or BMP signalling pathway (BMP4 and Noggin) (Figure 4D). The distribution of E-cadherin (E-cad) in interfollicular epidermis is similar in control and *Lhx2^Neo/Neo^* skin at E16.5 (Figure 4D). In summary, the use of a hypomorphic loss-of-function model for *Lhx2* has allowed us to follow HF development to a much later stage of embryonic development compared to *Lhx2^−/−^* embryos. By using this novel loss-of-function model we have been able to demonstrate that *Lhx2* is also required for HF morphogenesis, and the significant reduction in number of HFs suggests that *Lhx2* also play a role in HF induction.

### Lhx2 expression is sufficient to induce anagen

It has been previously reported that over-expression of *Lhx2* in early embryonic epidermis has no effect on HF morphogenesis [27]. This could be explained by the already high level expression of endogenous *Lhx2* in the developing HF (Figure 1A–1E), upon which additional *Lhx2* expression would have little effect. We therefore reasoned that investigation of the function of *Lhx2* in HFs would be helped by analysing the effect of transgenic expression of *Lhx2* when the endogenous gene is turned off during telogen (Figure 1J). To address this issue we developed a mouse model where we could induce Lhx2 expression in the epidermal portion of postnatal HFs. We utilised an expression system based on the Z/AP double reporter vector [39], where a floxed allele of *β-Geo* (encoding a β-galactosidase-Neomycin fusion protein) is followed by an expression cassette consisting of the *Lhx2* cDNA, an internal ribosomal entry site (IRES) and green fluorescent protein (GFP) cDNA (Figure S7A). A *Z/Lhx2-GFP* founder mouse strain with high β-Galactosidase (β-Gal) activity in the epithelial part of HFs during telogen and in ORS cells during anagen was chosen for further breeding (Figure S7B and S7C). Thus, DNA recombination by the Cre recombinase (e.g. following CreER expression and Tx treatment) will delete the *β-Geo* gene and induce expression of *Lhx2-GFP* primarily in the β-Gal^+^ cells (Figure S7).

**Figure 5:**
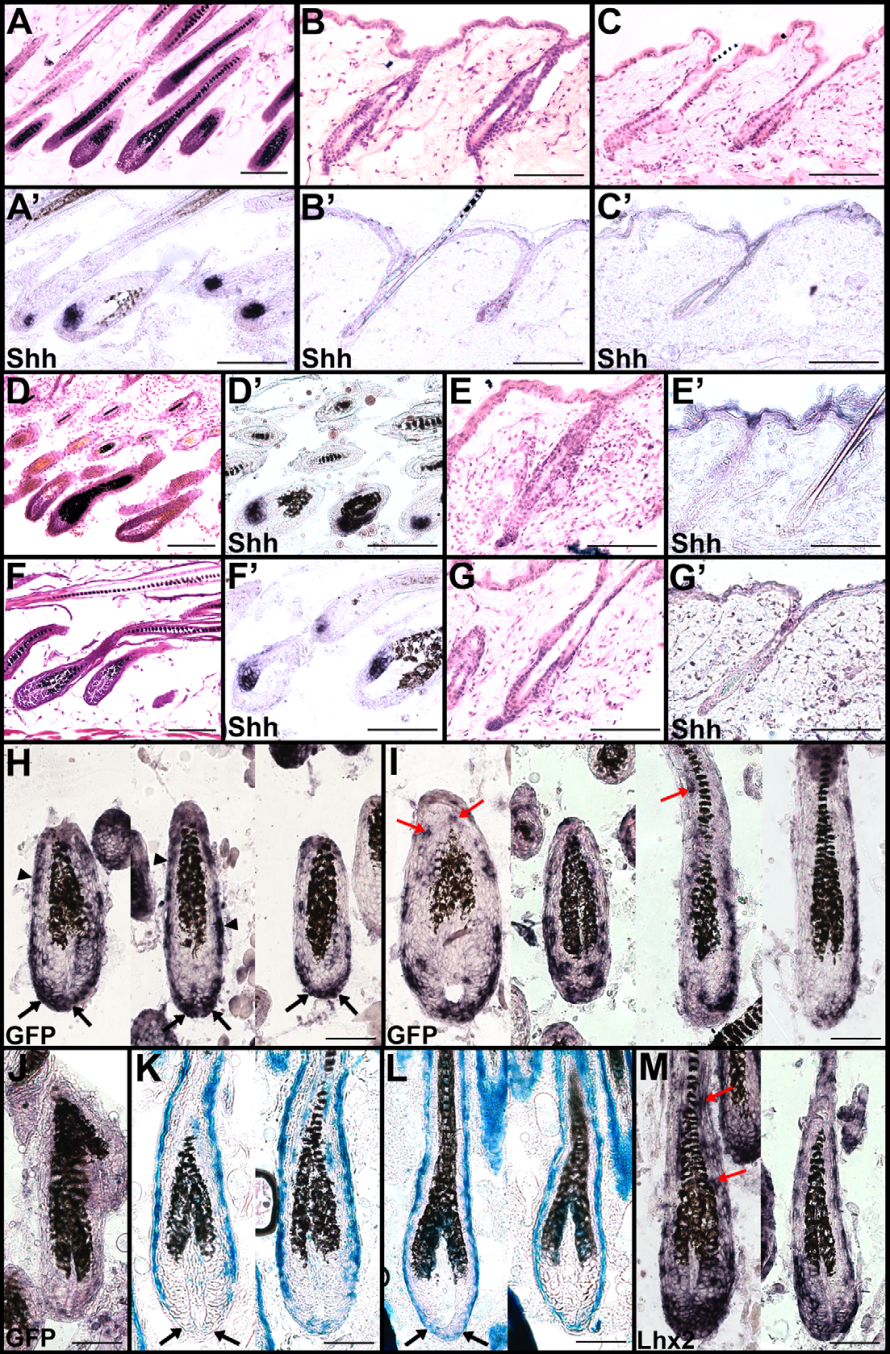
Lhx2 expression is sufficient to induce the anagen stage of the HF cycle. (A,A’) H/E staining of back skin HFs in anagen in 9 weeks old Tx-treated *Z/Lhx2-GFP:CreER* mice (A) revealing Shh expression (A’). (B,B’) H/E staining of back skin HFs in telogen in 9 weeks old Tx-treated control mice (B) revealing no Shh expression (B’). (C,C’) H/E staining of back skin HFs in telogen (C) retrieved from an area outside of the Tx-treated area in *Z/Lhx2-GFP:CreER* mice revealing no Shh expression (C’). (D–G,D’–G’) Representative examples of back skin HFs in 8 week and 2 day old Tx-treated *Z/Lhx2-GFP:CreER* mice where HFs in one individual are in anagen (D,D’) and in telogen in another individual (E,E’) showing that these mice also could enter telogen at the same time as the control animals (G,G’). The HFs were in anagen also in a slightly older Tx-treated Z/Lhx2-GFP:CreER animal (8-week- and 4-day-old) (F,F’). (H–J) In situ hybridization analyses for GFP expression in HFs from Tx-treated *Z/Lhx2-GFP:CreER* animals (H,I) and *Z/Lhx2-GFP* control mice (J). Most common expression pattern of GFP is in the proximal part of the hair bulb (H, black arrows) and in the ORS (H, arrow heads). GFP expression was also observed frequently in cells in the IRS (I, red arrows). (K,L) β-Gal staining of HFs in a Tx-treated *Z/Lhx2-GFP:CreER* (K) compared to HFs in a *Z/Lhx2-GFP* control animal (L). Lack of β-Gal^+^ cells in the proximal part of the hair bulb is in agreement with that most of the GFP^+^ cells are located at this part of the HF (black arrows). Control HFs contain numerous β-Gal^+^ cells in this area (L, black arrows). (M) Expression of Lhx2 analysed by in situ hybridization in HFs in Tx-treated *Z/Lhx2-GFP:CreER* mice. Expression is detected in all HFs and also in the IRS where Lhx2 is not expressed in control animals (red arrows). Scale bars: (A–G,A’–G’) 100 µm, (H–M) 50 µm.

To analyse the effect of Lhx2 expression on HF cycling we applied Tx onto shaved back skin of 5 week old control mice and *Z/Lhx2-GFP:CreER* double transgenic mice and analysed the mice at 8–9 weeks of age when HFs should be in telogen (Figure S2). Since most animals will complete an anagen-catagen-telogen transition between the Tx treatment and the time of analysis (Table S1; Figure S2), this experimental approach would avoid confounding effects of the shaving procedure and/or of the Tx treatment. Moreover, this approach allowed us to analyse if transgenic *Lhx2* expression influence the transition from anagen to telogen, and how it affects HFs in the extended telogen phase. HFs in all Tx-treated *Z/Lhx2-GFP:CreER* mice were in anagen at 9 weeks of age as determined by size, morphology, melanin deposition and Shh expression (Figure 5A and 5A’; Table 1), whereas HFs in almost all control mice were in telogen as expected at this age (Figure 5B and 5B’; Table 1; Table S1). Moreover, HFs that were outside of the Tx-treated area on *Z/Lhx2-GFP:CreER* mice remained in telogen (Figure 5C and 5C’).

**Table 1:** Lhx2 expression induce anagen.

Genotype	Age	Anagen	Telogen
Control	9 w.	3% (1/34)	97% (33/34)
*Z/Lhx2-GFP:CreER*	9 w.	100% (6/6†)	0% (0/6)
Control	8 w. 2–4 d.	0% (0/7)	100% (7/7)
*Z/Lhx2-GFP:CreER*	8 w. 2–4 d.	67% (2/3)	33% (1/3)
Control*	All	2% (1/41)	98% (40/41)
*Z/Lhx2-GFP:CreER*	All	89% (8/9‡)	11% (1/9)

*Genotypes of the control mice include non-transgenic (WT), *Z/Lhx2-GFP* single transgenic and *CreER* single transgenic animals. These control mice are also included in Table S1.

**†:** Chi-square analysis *Z/Lhx2:CreER* vs. control: p<0.001. Equal numbers of females and males in this group.

**‡:** Chi-square analysis *Z/Lhx2:CreER* vs. control: p<0.001

An extension of the growth phase of the HF by one week will generate noticeably longer hair in mice [40]. If our experimental set-up maintained anagen from Tx-treatment until the time of analysis, the length of anagen would have been extended by >2 weeks and hence lead to considerably longer hair. However, we did not observe any obvious increase in hair length in the Tx-treated *Z/Lhx2-GFP:CreER* mice (data not shown), suggesting that our experimental approach did not prolong the anagen phase but rather prematurely induced anagen in the following extended telogen. To further address this issue we analysed HFs in Tx-treated double transgenic mice closer to the time for anagen-catagen-telogen transition to elucidate whether animals expressing transgenic Lhx2 could enter telogen. At this time (8 weeks and 2–4 days old) the HFs were in telogen in one animal (Figure 5E and 5E’, Table 1) whereas the remaining mice had the HFs in anagen (Figure 5D, 5D’, 5F, and 5F’; Table 1). The HFs in all control mice for these time points were in telogen (Figure 5G and 5G’; Table 1). Thus, HFs where Lhx2 expression had been induced were able to enter telogen at the same time as control HFs at approximately 7–8 weeks of age, showing that the former HFs prematurely initiated anagen as they all were in anagen by 9 weeks of age (Table 1). Thus, the HFs in 89% (8/9) of the Tx-treated *Z/Lhx2-GFP:CreER* mice were in anagen, whereas HFs in 98% of the treated control animals were in telogen as expected (Table 1). The only time we observed HFs in telogen in Tx-treated *Z/Lhx2-GFP:CreER* mice was when analysed at an earlier time point close to the expected time for the anagen-catagen-telogen transition, suggesting that our experimental approach lead to premature initiation of anagen.

To confirm Cre-mediated recombination of the *Z/Lhx2-GFP* transgene and expression of Lhx2-GFP we analysed β-Gal activity and GFP expression in HFs in Tx-treated *Z/Lhx2-GFP:CreER* mice and control mice. Cells with robust expression of GFP were usually enriched at the proximal part of the hair bulb in 50% of the HFs in Tx-treated double transgenic mice (Figure 5H) whereas no GFP expression was detected in HFs in Tx-treated *Z/Lhx2-GFP* controls (Figure 5J). Numerous cells expressing endogenous Lhx2 were also located in the proximal part of the bulb during anagen (Figure 1G and 1H), suggesting that the cells expressing transgenic Lhx2 follow the same migration path as the cells expressing the endogenous gene. GFP was also expressed by cells in the ORS, which is in agreement with the distribution of the β-Gal^+^ cells in *Z/Lhx2-GFP* control mice (Figure 5H and 5L; Figure S7C). Although most HFs showed GFP expression in the proximal part of the hair bulb the distribution and number of GFP^+^ cells in the remaining HFs varied considerably (Figure 5I). Both GFP^+^ and Lhx2^+^ cells were frequently detected in the IRS (Figure 5I and 5M), showing that follicular progenitor cells can migrate into the IRS despite maintained expression of *Lhx2*. However, Cre-mediated excision of *β-Geo* was not complete in the Tx-treated *Z/Lhx2-GFP:CreER* mice since numerous β-Gal^+^ cells were present in these HFs (Figure 5K). As expected, GFP expression and β-Gal activity appears to be mutually exclusive since cells lacking β-Gal activity were enriched in GFP^+^ regions in the proximal part of the bulb (Figure 5K and 5H), whereas numerous β-Gal^+^ cells are located in this region in control *Z/Lhx2-GFP* mice (Figure 5L and Figure S7C). Some HFs in Tx-treated *Z/Lhx2-GFP:CreER* mice contained more β-Gal^+^ cells which is in agreement with the decreased number of GFP^+^ cells in some HFs (Figure 5K and 5I). Since Lhx2 expression is detected in all HFs in these mice whereas expression of GFP is not (compare Figure 5M to Figure 5H and 5I), indicates that the endogenous *Lhx2* gene is also expressed as in normal anagen. Thus, relatively few cells expressing the Lhx2 transgene is sufficient to induce endogenous Lhx2 expression in a cell nonautonomous manner and thereby prematurely initiate anagen.

## Discussion

**Figure 6:**
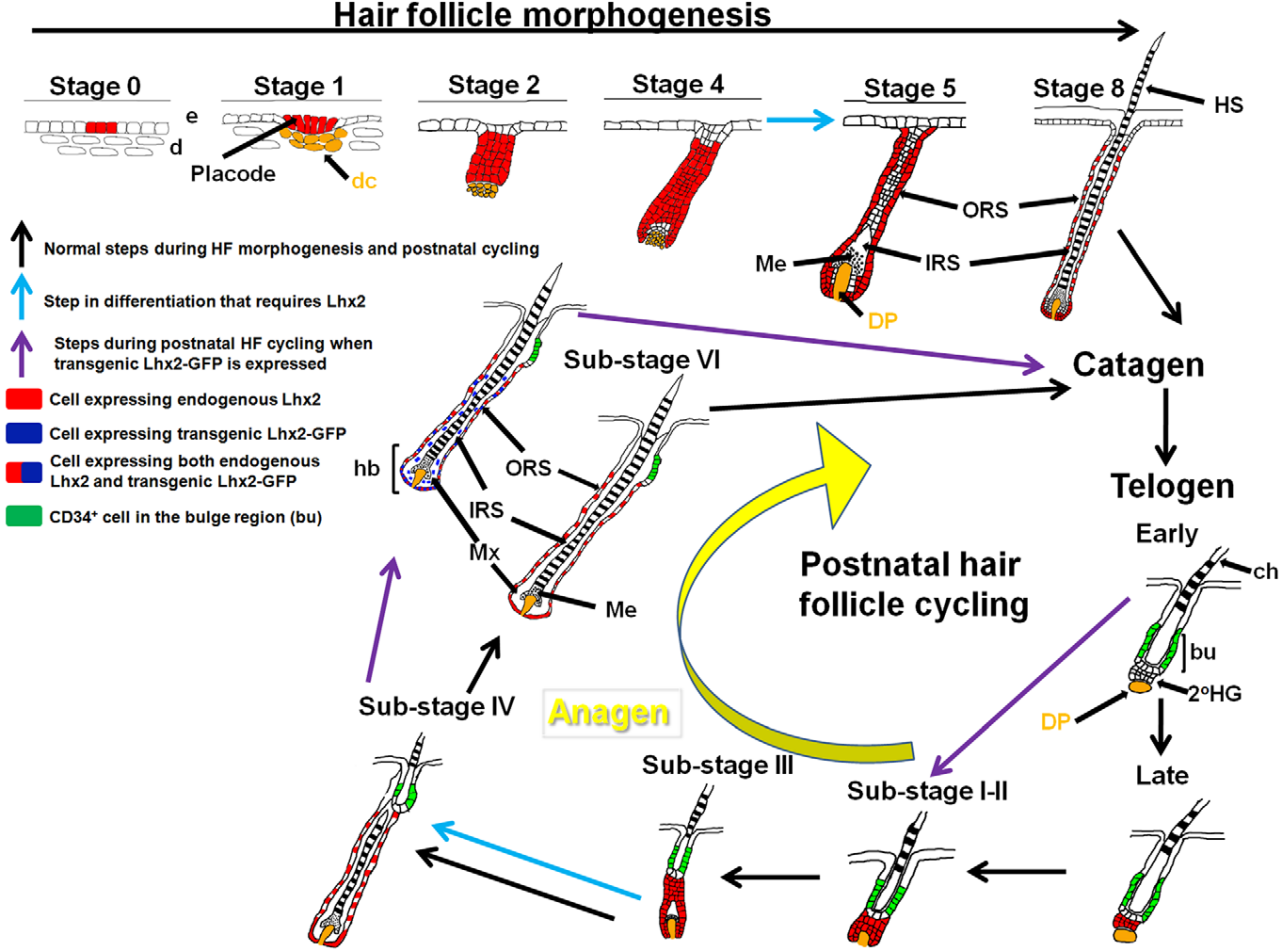
Overview of the expression pattern of Lhx2 and a model for the function of *Lhx2* in HF morphogenesis and postnatal HF cycling. Lhx2 is expressed from an early time point during HF morphogenesis in basal keratinocytes before a dermal condensate (dc) is formed (Stage 0), and is broadly expressed in the epithelial part of the HF in the subsequent stages of morphogenesis. In a fully developed HF (Stage 8 of morphogenesis), Lhx2 is expressed by cells in the ORS and in the most proximal part of the hair bulb (hb). When the HF enters telogen Lhx2 expression becomes undetectable (Telogen, Early) but expression reappears in CD34^−^ cells in the secondary hair germ (2°HG) immediately prior to anagen induction (Telogen, Late). The pattern of Lhx2 expression during morphogenesis is thereafter reiterated during anagen progression (Sub-stages I-VI) until the next anagen-catagen-telogen transition occurs and Lhx2 expression is turned off. The conditional inactivation of Lhx2 in postnatal HFs reveal that these HFs do not develop beyond Sub-stage III of the anagen phase showing that Lhx2 is required to develop further (blue arrow in anagen progression). The mouse strain homozygous for the hypomorphic allele of *Lhx2* (*Lhx2^Neo/Neo^*), revealed that embryonic HFs are developmentally arrested at an early stage in this loss-of-function model. Since we rarely identified HFs at Stage 5 we suggest that Lhx2 is required to develop beyond Stage 4 (blue arrow in HF morphogenesis). HFs expressing transgenic *Lhx2* (Lhx2-GFP) during postnatal HF cycling can enter catagen and telogen similar to control HFs, but transgenic Lhx2 expression causes premature initiation of anagen (purple arrows). The cells expressing transgenic Lhx2 preferentially locate to the proximal part of the hair bulb where the cells expressing endogenous Lhx2 are also located. Since transgenic expression of Lhx2 appears to induce endogenous Lhx2 expression, we suggest that numerous cells co-express the transgene and the endogenous gene, particularly in the proximal part of hb. Cells expressing transgenic Lhx2 are also located in the IRS whereas endogenous Lhx2 is not expressed in this area. 2°HG, secondary hair germ; bu, bulge region; ch, club hair; d, dermis; dc, dermal condensate; DP, dermal papilla; e, epidermis; hb, hair bulb; HS, hair shaft; IRS, inner root sheath; Me, melanocytes synthesising pigment; Mx, matrix; ORS, outer root sheath.

We have analysed the function and expression of the LIM-homeobox gene *Lhx2* in HFs during morphogenesis and the cyclic phases of postnatal hair growth (summarised in Figure 6). *Lhx2* is expressed in basal keratinocytes prior to the formation of the hair placode at initiation of morphogenesis. Expression becomes restricted to matrix cells at the proximal part of the hair bulb and to cells in the ORS in a fully developed HF. When the HF enters telogen Lhx2 expression becomes undetectable. However, during late telogen Lhx2 expression re-appears in CD34^-^ cells located in the secondary HG and this expression is maintained when anagen commences. The expression observed during morphogenesis is thereafter reiterated during progression of anagen. This developmental and stage-specific expression pattern suggests that Lhx2 is involved in the generation and regeneration of hair. This hypothesis is supported by mouse models where Lhx2 expression could either be conditionally inactivated, significantly reduced, or switched on in the HF. The loss-of-function mouse models revealed that Lhx2 is required for progression of both anagen and morphogenesis and hence hair formation. The gain-of-function mouse model showed that Lhx2 expression is sufficient to induce anagen. The function of Lhx2 in the HF has been addressed previously [27], concluding that Lhx2 is important for maintaining the quiescence of the stem cells located in the bulge region. This interpretation of Lhx2 function is difficult to reconcile with the expression pattern and the phenotypes in the gain-of-function and loss-of-function mouse strains reported herein. The reasons for these discrepancies in interpretations of the function of Lhx2 in the HF are not clear. We do, however, detect Lhx2 protein in cells in the proximal part of the bulge region, which is in agreement with Rhee *et al.* [27], although we are unable to detect Lhx2 mRNA in these cells. The functional relevance of this observation remains to be elucidated. The different interpretation of the function of Lhx2 could be explained by the different approaches used to study gain-of-function and loss-of-function. Rhee *et al*. [27] expressed Lhx2 under the control of the keratin-14 promoter leading to epidermal Lhx2 expression early in embryonic development, probably prior to HF induction [41], and when the endogenous *Lhx2* gene is widely expressed in the developing HF. We induced expression in HFs when the endogenous gene is turned off during telogen. Rhee *et al*. analysed loss-of-function by transplanting HFs from *Lhx2^−/−^* embryos onto skin of nude mice, whereas we analysed intact postnatal HFs where the *Lhx2* gene had been conditionally inactivated. Rhee *et al*. also showed a significantly reduced number of HFs in the conventional *Lhx2* mutant embryos, which is in agreement with our loss of function data in embryos of the same gestational age. However, since the *Lhx2^−/−^* embryos start to die *in utero* even prior to initiation of HF morphogenesis, we confirmed these observations in a mouse strain harbouring a hypomorphic allele of Lhx2 (*Lhx2^Neo^*) where HF development can be followed to a gestational age when pelage HF morphogenesis is firmly established.

The Bulge Activation hypothesis has been the dominating theory of induction of anagen at the expense of the HF Predetermination hypothesis. The Bulge Activation hypothesis suggests that stem cells in the bulge region start to proliferate after responding to signal(s) emanating from the DP, generating transient amplifying cells that migrate to the secondary HG and initiate anagen [5],[42]. The HF Predetermination hypothesis suggests that the secondary HG itself contains stem cells that start to proliferate after receiving signals from the DP without input of transient amplifying cells from the bulge region [43],[44]. This hypothesis has been discarded mainly due to the failure to detect slowly dividing cells, or so-called label-retaining cells (LRCs), in the secondary HG. The observation that Lhx2 expression is initiated in CD34^−^ cells in the secondary HG, and not in CD34^+^ cells in the bulge region, just prior to and at initiation of anagen fits with the HF Predetermination hypothesis. Although we cannot exclude that a few CD34^+^Lhx2^+^ cells might be present in the bulge, the majority of the Lhx2^+^ cells are CD34^−^ and are located in the secondary HG, the ORS, and in cells in the proximal part of the hair bulb. Thus, our data strongly suggest that Lhx2 expressing cells are involved in the expansion and patterning of the transient portion of the HF. An alternative interpretation that would fit with the Bulge Activation hypothesis is that Lhx2^+^ cells in the secondary HG in late telogen relay signal(s) from the DP to the cells in the bulge region leading to the migration of these cells into the secondary HG and hence initiation of anagen. Consequently, the stem cells start to express Lhx2 when they have left the bulge region and entered the secondary HG. Thus, irrespective of which hypothesis prevails, the Lhx2^+^ cells in the HG represent an epidermal progenitor/stem cell population that is distinct from those in the bulge region.

The CD34^+^ cells in the bulge region are LRCs and many of their stem cell properties have been based on this feature [5],[31], whereas no LRCs are present in the secondary HG [5],[43]. We show that Lhx2 is primarily expressed by CD34^−^ cells and hence Lhx2 is most probably not expressed by LRCs in the bulge region. However, a recent study where the fate of cells expressing the leucine-rich G protein-coupled receptor 5 (Lgr5) was analysed, question the link between stem cells and LRCs in HFs [45]. The Lgr5^+^ cells were suggested to define a novel stem cell population that cycles, is long lived and multipotent, contradicting the generally held view that “true” stem cells are quiescent and hence LRCs, whereas their progeny (i.e. transient amplifying cells) actively cycle and are short-lived. The authors suggest that Lgr5 expression defines a novel stem cell population distinct from the CD34^+^ LRCs in the bulge region and argue for a reinvestigation of the relationship between HF stem cells and LRCs. The expression pattern of this novel stem cell marker in the HF is very similar to that of Lhx2 since both Lgr5 and Lhx2 are expressed in the secondary HG, in the ORS and in matrix cells in the proximal part of the hair bulb. A significant overlap in Lhx2 and Lgr5 expression is also corroborated by Jaks *et al.* [45] who show that Lhx2 is only expressed by Lgr5^+^ cells in the HF. Although the exact relationship between Lhx2 and Lgr5 expression at the cellular level remains to be elucidated, the respective expression pattern suggests that they are highly overlapping and hence defines a similar, if not an identical, cell population. Our data also suggest that Lhx2 expression during late telogen might be an “activation marker” of the putative Lgr5^+^CD34^−^ follicular stem/progenitor cell population in the secondary HG, and that the Lhx2^+^Lgr5^+^CD34^−^ cells initiate the anagen phase. Moreover, recent data also suggests that cells in the secondary HG become activated during late telogen prior to anagen induction which is also accompanied by changes in gene expression pattern in the DP [46], and the expression of Lhx2 might be a part of this process. The finding that over-expression of Lhx2 in the epidermal part of the HF is sufficient to prematurely induce anagen is in agreement with this hypothesis. However, Lhx2 is not required for anagen (or HF morphogenesis) induction whereas it is required for the progression of anagen and morphogenesis, revealing that redundant mechanism(s) can compensate for Lhx2 in the initiation process. The molecular basis of this redundancy in the initiation of anagen is unknown, but a role of other LIM homeobox genes is unlikely since the related LIM-homebox genes *Lhx9* and *Lmx1b*, which can compensate for Lhx2 in limb development [47], are not expressed in follicular epidermis (unpublished data). It remains to be elucidated whether or not upregulation of Lhx2 expression during late telogen is an intrinsic property of the epidermal progenitor cells, or a consequence of signal(s) emanating from the DP, and/or linked to cyclic BMP signalling regulating stem cell activation [48],

Relatively few cells express the Lhx2 transgene in HFs of Tx-treated *Z/Lhx2-GFP:CreER* mice, which might explain why the HFs can enter telogen phase similar to the control HFs. A more robust expression of transgenic Lhx2 might give a different outcome, and this issue is being addressed at present. Moreover, the ability of cells expressing transgenic Lhx2 to migrate in the same manner as cells expressing endogenous Lhx2 might also explain why HFs prematurely initiate a new anagen phase. Thus, cells expressing transgenic Lhx2 would be close to the DP and convey epidermal signals to it, similar to the activation of the HG cells prior to anagen initation [46]. Consequently the DP would signal back to epidermal cells since continuous signalling between epidermal cells and DP is required for hair formation [2],[10]. This could, in part, explain why relatively few Lhx2 expressing cells in postnatal HFs can induce endogenous Lhx2 expression in a cell nonautonomous manner and initiate anagen prematurely. Thus, in this mouse model initiation of anagen is promoted whereas the duration of anagen is not affected.

The Lhx2 loss-of-function models have revealed that Lhx2 is necessary for the progression of both anagen and morphogenesis. Since the Hedgehog, Wnt and Nog/BMP signalling pathways are important for anagen and morphogenesis initiation/progression [11]–[14],[17],[18], we analysed whether any of these pathways were affected in the mutant HFs. However, no difference in expression of the central mediators of Nog/BMP, Hedgehog (Shh, Smo, Ptc1) or Wnt (Lef1) signalling could be observed between control and mutated HFs. Furthermore, activation of the respective pathways in mutant HFs was supported by the presence of the stabilized form of β-catenin that mediates Wnt signalling [49], expression of Ptc1 that is a downstream transcriptional target gene in Hedgehog signalling [11],[50], and expression of Lef1, which is induced by Nog [51]. Signalling via NF-κB induced by the Tumour Necrosis Factor (TNF) family of ligands is also an important pathway promoting HF morphogenesis [52]. Although we have not directly analysed this pathway in mutant HFs, it is unlikely that Lhx2 is involved in this pathway since active NF-κB signalling induces expression of Shh [53], which is clearly expressed in both the conditional mutant as well as the hypomorphic mouse strain. Thus, as far as we can determine, loss of Lhx2 function does not disturb any major signalling pathway important for anagen initiation/progression or HF morphogenesis. Moreover, significant numbers of proliferating cells are present in the mutated HFs showing that the developmental arrest at anagen Sub-stage III cannot solely be due to lack of proliferating cells at this stage. Co-factor of LIM domain 2 (Clim2) is expressed in the ORS and the matrix cells and has been shown to physically interact with Lhx2 [54]. Transgenic expression of a dominant negative Clim in the HF using a K14 promoter leads to a progressive hair loss during postnatal life due to aberrant HF differentiation and disrupted HF structure [54], revealing that other means to interfere with Lhx2 function also hampers hair formation. These data suggest that Lhx2 play a role in the differentiation/patterning of the HFs. Such a function of Lhx2 has previously been shown in the fetal liver where Lhx2, both in a cell autonomous and cell nonautonomous manner, is involved in the expansion, differentiation and organisation of all cellular components of the liver into a functional 3-dimensional structure [25]. The exact mechanism(s) by which Lhx2 can regulate such complex processes in organ development is a central question in organogenesis.

Our results show that Lhx2 can regulate two separable processes in HF cycling; firstly, Lhx2 can initiate anagen but it is not required for this process, secondly, Lhx2 is required for the progression of anagen (and morphogenesis) generating a fully assembled hair shaft. These observations indicate a rather complex role for Lhx2 in HF biology, suggesting that Lhx2 can initiate the signalling cascades necessary for anagen initiation, as well as regulate the process of anagen progression and hence hair formation. The significantly reduced number of HFs in the mouse strain homozygous for the hypomorphic allele of *Lhx2* as well as in the *Lhx2^−/−^* mice as previously reported [27], further supports the idea that Lhx2 has an important function during the early stages of HF development. We have not yet been able to determine the molecular basis for Lhx2 activity in the HF. However, we have previously performed a global gene expression analysis of hematopoietic stem cell-like cell lines with inducible Lhx2 expression where we could identify 141 genes that correlated to Lhx2 expression. Gene Ontology classification of these genes revealed that genes related to ‘regulation of signal transduction’ and ‘organogenesis’ were over-represented [26]. A considerable fraction of these genes (31%) that we have analysed are expressed in the HF, for example *Nuak1* (NM_001004363), *Tmem2* (NM_031997), *Etv5* (NM_023794), and *Enc1* (NM_007930) [26], suggesting a putative overlap in Lhx2 function between different tissues. Further elucidation of the molecular basis of Lhx2 function in the HFs is important to fully understand the complex regulation of hair formation.

## Materials and Methods

### Ethics statement

All experiments involving animals were approved by the Animal Review Board at Umeå University.

### Staging of HFs and timing of the synchronised postnatal hair cycles on back skin

Staging of hair follicle morphogenesis is according to [28], and the staging and Sub-stages of postnatal HF cycles and the nomenclature of HFs used throughout this work are according to [29]. The initial postnatal hair cycles of back skin HFs in female C57BL/6 mice are synchronized [29] (Figure S2). Since we have analysed both female and male mice with a mixed genetic background (C57BL/6 x 129/Sv), we determined the timing of the initial postnatal hair cycles in our mouse breeding stock used for these experiments to exclude any major deviations from what has been published (summarised in Table S1). The timing of the first postnatal telogen-anagen transition in 3–4 weeks old mice and the first postnatal anagen-catagen-telogen transition in 6–8 week old mice agreed fairly well with previous findings (Table S1; Figure S2) [29]. Moreover, the HFs on the back skin of most control mice had entered the second and extended telogen at 7–8 weeks of age (Table S1). According to [29] (Figure S2), the second postnatal anagen is initiated at 12 weeks of age but we were unable to define a synchronized starting point of the second postnatal anagen in our control animals (Table S1).

### Generation and maintenance of mice and tamoxifen-treatment

All mice were maintained at the animal facility at Umeå University under pathogen-free conditions. Generation of the Z/Lhx2-GFP transgenic mouse strain was essentially as described previously for the Z/AP double reporter mouse strain [39], but the AP part in the Z/AP double reporter vector was replaced by Lhx2-GFP to generate the Z/Lhx2-GFP vector. E14 ES cells were electroporated (Bio-Rad GenePulser) with the Z/Lhx2-GFP vector and G418-resistent cells were selected in 350 µg/mL of G418 (GIBCO, 11811-031). ES cell clones were isolated and those showing the highest β-Gal activity and had a single integration of the construct was further selected and used for blastocyst injections at the Umeå Transgene Core Facility (UTCF). Blastocysts were transferred to pseudo-pregnant females and chimeric offspring were crossed with C57BL/6 mice to obtain germ-line transmission of the *Z/Lhx2-GFP* transgene. The *Z/Lhx2-GFP* mouse strain with the highest β-Gal activity in HFs was chosen for further experiments (Figure S7B and S7C).

The mouse strain with a hypomorphic allele of Lhx2 (*Lhx2^Neo^*) was generated by inGenious Targeting Laboratory Inc. (www.genetargeting.com). The presence of the Neo gene in the intact Lhx2 locus between exon 2 and 3 in the opposite transcriptional orientation was confirmed by Southern blot analyses on ES cells and by PCR on tail biopsies (Figure S4A). Mice containing a floxed allele of Lhx2 were obtained by crossing *Lhx2^Neo/+^* mice with mice transgenic for the Flp recombinase as this will generate offspring where Neo is deleted and leave two *loxp* sites flanking exon 2 (Figure S4B). We have also confirmed the phenotype using another *Lhx2^flox^* mouse strain where exons 2 and 3 are flanked by *loxp* sites [32], which are the exons deleted in the conventional knock-out [22]. The mutated Lhx2 gene is expressed but Lhx2 mRNA missing either exon 2 or exons 2 and 3 are unable to make Lhx2 protein containing any functional domains since both transcripts are out-of-frame down-stream of exon 1.

The *Z/Lhx2-GFP* and *CreER* [33] transgenes, and the *Lhx2^Neo^* and *Lhx2^flox^* alleles were maintained on a mixed genetic background (C57BL/6 X 129/Sv). The *Lhx2^flox/flox^* and *Lhx2^flox/-^* mice are healthy and fertile. Primers used to identify the *Lhx2^Neo^* and *Lhx2^flox^* alleles were: DL1 5′-GTTCTAGAAGTGGAAGGGGAGTGG-3′, LOX 5′-GCCAGACTAGCAGACGCTGC-3′, SDL2 5′-CCACCGGTACTCCTCTTCAGAG-3′, UNI 5′-AGCGCATCGCCTTCTATCGCCTTC-3′, AT1 5′-CACTCCGAGCCTGTTTGGTG-3′. Primers used to identify the Z/Lhx2-GFP transgene were GFPforward 5′-TTCCACCATATTGCCGTC-3′ and GFPreverse 5′-AGAACTTGCCGCTGTTCA -3′.

The morning of the vaginal plug was considered as E0.5. Dorsal (back) skin was shaved with electric razor and 1 mg of 4-hydroxytamoxifen (Sigma H7904) dissolved in acetone was applied to the shaved area once a day for 7–9 consecutive days.

### Histology, in situ hybridization, β-gal staining, alkaline phosphatase staining, and immunofluoresence

Skin was isolated and fixed in 4% paraformaldehyde (PFA) in PBS at 4°C, transferred to 30% sucrose in PBS for 24 hours at 4°C, mounted in Tissue-Tek (Sakura) and stored at −80°C. Sectioning (8 µm) was performed in a cryostat (Microm HM505E) and collected on Superfrost Plus slides (Menzel-Gläser). For hematoxylin-eosin staining tissue sections were incubated in Mayer’s hematoxylin solution for 2 min, water 15 min, eosin solution 2 min, 95% ethanol 2×1 min, 99% ethanol 2×1 min and in xylene for 5 min. The slides were mounted with DPX mounting media (VWR).

For β-Gal staining skin tissue fixed for 30 min in PFA was cryosectioned, washed for 3×20 min in wash buffer (0.1M phosphate buffer, 2 mM MgCl_2_, 5 mM EGTA, 0.02% NP40 and 0.01% sodium deoxycholate) and subsequently incubated in wash buffer supplemented with 1 mg/ml 5-bromo-4-chloro-3-indolyl-D-galactopyranoside (X-gal, Austral), 5 mM potassium ferrocyanide and 5 mM potassium ferricyanide, for 4–8 hours at room temperature. The reaction was stopped with 3×5 min washes with PBS, and sections were mounted in 87% glycerol.

In situ hybridization using DIG labelled probes was performed essentially as described previously [55],[56]. Skin fixed in PFA for 24 hours was sectioned and treated with 10 µl/ml proteinase K (Roche) in 0.1 M PBS for 15 min at room temperature prior to hybridization. The DIG-signal was visualized with AP-conjugated anti-DIG Fab_2_ fragments and developed using NBT and BCIP (Roche). The following probes were used: Lhx2 (NM_010710, full length cDNA nucleotides 460–1750, exon 2 nucleotides 587–789, exon 2–3 nucleotides 643–1172), GFP (hrGFP, complete coding region), Shh (NM_017221, nucleotides 1–1715), Smo (NM_176996, nucleotides 2957–3813), Ptc1 (NM_008957, nucleotides 1–841), Lef1 (BC057543, nucleotides 1678–2999), BMP4 (NM_007554, nucleotides 117–578), and Hist1h3c (NM_175653, nucleotides 27–480).

Immunohistochemistry was performed essentially as previously described [57]. Skin fixed for 30 min or 2 hours was sectioned and slides were washed 3×5 min in TBS (50 mM Tris-HCl pH 7.4, 150 mM NaCl) and blocked with 10% FCS in TBST (TBS with 0.1% Triton X-100) for 20 min. The primary antibodies rat anti-CD34 (MEC 14.7, Abcam, dilution 1:50) and rat anti-E-cadherin (Zymed Laboratories Inc., dilution 1∶300) were applied to slides over night at 4°C diluted in TBST with 5% FCS. After 3×5 min washing in TBST, the secondary antibody, donkey anti-rat IgG labelled with Alexa Fluor 488 (Molecular Probes, dilution 1∶400), was added together with DAPI for 1 hour at room temperature. The primary rabbit anti-mouse Lhx2 (generous gift from Dr. Sara Wilson, dilution 1∶32,000) was applied to slides over night. After 3×5 min washing in TBST, the secondary antibody, Cy3-conjugated donkey anti-rabbit IgG (Jackson ImmunoResearch Laboratories Inc., dilution 1∶400), was added together with DAPI for 1 hour at room temperature. Slides were subsequently washed 3×5 min in TBST before mounting with fluorescence mounting medium (Vectashield, Vector Laboratories). For the immunolabelling of the active (dephosphorylated) form of β-catenin, skin fixed for 2 hours was used. Slides were washed 3×5 min in TBST, blocked for endogenous peroxidase activity in 2% H_2_O_2_, 80% methanol for 20 min, washed, incubated in citrate buffer (10 mM sodium citrate, pH 6.0) at 95°C for 20 min for epitope retrieval and washed. After blocking with 10% FCS in TBST for 1 hour, the primary mouse anti-β-catenin antibody (Upstate 05-665, dilution 1∶400, recognise non-phosphorylated Ser-37 and Thr-41) was applied to the slides over night at 4°C. After washing, the slides were exposed to the secondary biotinylated anti-mouse antibody (dilution 1∶400) for 2 hours at room temperature, washed and incubated for 1 hour in Avidin-Biotin Complex (ABC) solution (Vector; 2% buffer A, 2% buffer B and 10% FCS in TBST), washed 3×5 min in TBST and developed in DAB-solution (Sigma) with 0,01% H_2_O_2_ for 5 min. After thorough washing in cold PBS, the slides were mounted in 87% glycerol.

Skin fixed in PFA was used for detection of AP activity. The sections were fixated in acetone at −20°C for 5 min, washed for 3×5 min in PBS, rinsed 5 min in TN buffer pH 9.5 (0.1 M Tris-HCl, 0.1 M NaCl) and exposed to 125.6 µg/ml NBT and 63 µg/ml BCIP in TN buffer for 20 min in darkness. Sections were washed with PBS 3×5 min to stop the reaction and subsequently mounted in 87% glycerol.

The number of HFs in embryos was calculated from two sections from two different control embryos and two sections from two different *Lhx2^Neo/Neo^* embryos at E16.5, and four sections from two control animals and two sections from two *Lhx2^Neo/Neo^* embryos at E18.5.

### Statistical analyses

Statistical analyses were carried using Student’s t-test or the Chi-square method.

## Supporting Information

Figure S1 Lhx2 expression in whisker HFs mimics the expression pattern in pelage HF. (A) In situ hybridization analysis reveals *Lhx2* expression in the ORS (arrow heads) and in matrix cells in the proximal part of the hair bulb (arrows) during whisker HF morphogenesis. (B) In situ hybridization analysis reveals *Lhx2* expression in the ORS (arrow heads) and in matrix cells in the proximal part of the hair bulb (arrows) in an adult whisker HF. Scale bar, 100 µm.
(1.41 MB TIF)

Figure S2 Time scale for the first postnatal and synchronized HF cycles in female C57BL/6 mice, and an overview of the strategy to conditionally inactivate the *Lhx2* gene or induce transgenic expression of Lhx2. The time scale to illustrate the temporal progression through the postnatal HF cycles is adapted from [29]. The *Lhx2* gene was conditionally inactivated by treating shaved back skin on *CreER:Lhx2^flox/flox^* and *CreER:Lhx2^flox/-^* mice with Tx during the first postnatal telogen phase in approximately 3-week-old mice (indicated by the first red bar between week 3 and 4). The progression of the first postnatal anagen phase was analysed in these mice and control *Lhx2^flox/flox^* and *Lhx2^flox/-^* animals at 5.5 to 6 weeks of age (indicated by the second red bar between week 5 and 6). To induce transgenic expression of Lhx2 in postnatal HFs the *CreER:Z/Lhx2-GFP* double transgenic mice were shaved on the back skin and treated with Tx in approximately 5 weeks old mice (indicated by the first yellow bar at 5 weeks). The effect of transgenic Lhx2 expression was analysed and compared to control single transgenic mice (*CreER* or *Z/Lhx2-GFP*) during the extended telogen phase at 8–9 weeks of age (indicated by the second yellow bar between week 8 and 9).
(0.10 MB TIF)

Figure S3 Over-view of Lhx2 expression pattern of HFs in early telogen, late telogen and early anagen Sub-stages I/II and III. (A) Analysis of Lhx2 expression in HFs in early telogen (3-week- and 1-day-old mice). Most mice at this age have their HFs in telogen (Table S1). No expression can be detected in any HF at this stage. (B) Analysis of Lhx2 expression in HFs in telogen in late telogen (3 week and 4 days old mice). Numerous mice have initiated anagen at this age (Table S1). Homogenous and distinct expression is detected in the secondary HG of all HFs at this stage (arrows). Shh is not expressed in HFs at this stage confirming that HFs are in telogen (Figure 2I). (C) Analysis of Lhx2 expression in HFs in anagen Sub-stages I-II (prior to pigment deposition). Lhx2 expression is detected in all HFs in the secondary HG and most epithelial cells in the down-growing part of the HF. Shh is expressed in all HFs at this stage (Figure 2M). (D) Analysis of Lhx2 expression in HFs in anagen Sub-stage III (all HFs contain pigment and most hair shaft has not reached the hair canal). Lhx2 expression is detected in cells in the ORS (arrow heads) and the proximal part of the hair bulb (arrows). Scale bar, 100 µm.
(4.19 MB TIF)

Figure S4 Description of the mouse strains with a hypomorphic allele of *Lhx2* (*Lhx2^Neo^*), and a floxed allele of *Lhx2* (*Lhx2^flox^*). (A) Description of the hypomorphic allele of *Lhx2* locus containing the *Neo* gene between exon 2 and 3 in the opposite transcriptional orientation (upper panel). The location of the primers used to identify the WT allele and the *Lhx2^Neo^* allele are indicated (upper panel) and the PCR results to identify mice that are WT, heterozygous or homozygous for the *Lhx2^Neo^* allele are shown (lower panel). *lox* sites are indicated by black triangles and *Flp* sites are indicated by white arrow heads. (B) Description of the “floxed” Lhx2 allele (*Lhx2^flox^*) after Flp-mediated deletion of the *Neo* gene (upper panel). The location of the primers used to identify the *Lhx2^flox^* allele are indicated (upper panel) and the PCR results to distinguish mice that have the *Lhx2^Neo^* allele or the *Lhx2^flox^* allele are shown (lower panel). (C) Comparison between an E18.5 control embryo (left) and an *Lhx2^Neo/Neo^* embryo (right). The *Lhx2^Neo/Neo^* embryos develop the same eyeless phenotype as the *Lhx2^−/−^* embryos confirming that Lhx2 expression is significantly decreased. However, the anemia in *Lhx2^Neo/Neo^* embryos is less severe compared to the *Lhx2^−/−^* embryos and the expected number of live embryos can therefore be obtained at E18.5 when pelage HF morphogenesis is well established.
(0.88 MB TIF)

Figure S5 Conditional inactivation of *Lhx2* is incomplete in Tx-treated *CreER:Lhx2^flox/flox^* mice leading to re-growth of some hair. In situ hybridization analyses on HFs from the shaved and Tx-treated area on *CreER:Lhx2^flox/flox^* mouse where hair started to re-grow. (A) In situ hybridization using the full length Lhx2 probe that detects both the WT allele and the mutant allele. (B) In situ hybridization using the probe restricted to exon 2 that only detects the WT allele (arrows), revealing incomplete inactivation of the *Lhx2* gene leading to rescue of hair growth.
(2.53 MB TIF)

Figure S6 The S-phase-specific histone gene *Hist1h3c* is only expressed during anagen. In situ hybridization analyses of *Hist1h3c* expression of HFs in telogen (A) and anagen (B). (C) *Hist1h3c* is expressed in anagen HFs where Lhx2 has been conditionally inactivated in a similar pattern as in control HFs.
(1.20 MB TIF)

Figure S7 Generation of a transgenic mouse strain where Lhx2 expression can be induced in postnatal HF epidermis. (A) Schematic representation of the vector used to generate the *Z/Lhx2-GFP* transgenic mouse strain (upper panel) and the organisation of this vector after Cre-mediated recombination (lower panel). The blue arrows correspond to the mRNA that is generated before and after Cre-mediated recombination of this vector. The expression system is based on the Z/AP double reporter vector developed by Lobe and co-workers [39], where a floxed allele of β-Geo (encoding a β-galactosidase-Neomycin fusion protein) is followed by an expression cassette consisting of the Lhx2 cDNA, an internal ribosomal entry site (IRES) and green fluorescent protein (GFP) cDNA. Thus, DNA recombination by the Cre recombinase will delete the *β-Geo* gene and place *Lhx2-GFP* immediately downstream of the promoter/enhancer leading to its transcription. We generated a mouse strain transgenic for this vector (*Z/Lhx2-GFP*) that showed β-Gal activity in a variety of tissues including epidermis and in the epithelial portion of HFs in telogen (B) as well as anagen (C). The *Z/Lhx2-GFP* mouse strain was crossed with the *CreER* transgenic mouse strain where Tx-treatment of skin of the *Z/Lhx2-GFP:CreER* double transgenic mice will lead to activation of Lhx2 expression in HFs.
(1.18 MB TIF)

Table S1 Hair cycle stage of back skin HFs in control mice at different ages.
(0.10 MB PDF)
