# Supplementary figures and images for: Cyclic Expression of Lhx2 Regulates Hair Formation
Source: PLoS Genet. 2010 Apr 8;6(4):e1000904. doi: 10.1371/journal.pgen.1000904 (PMC2851574; doi:10.1371/journal.pgen.1000904)

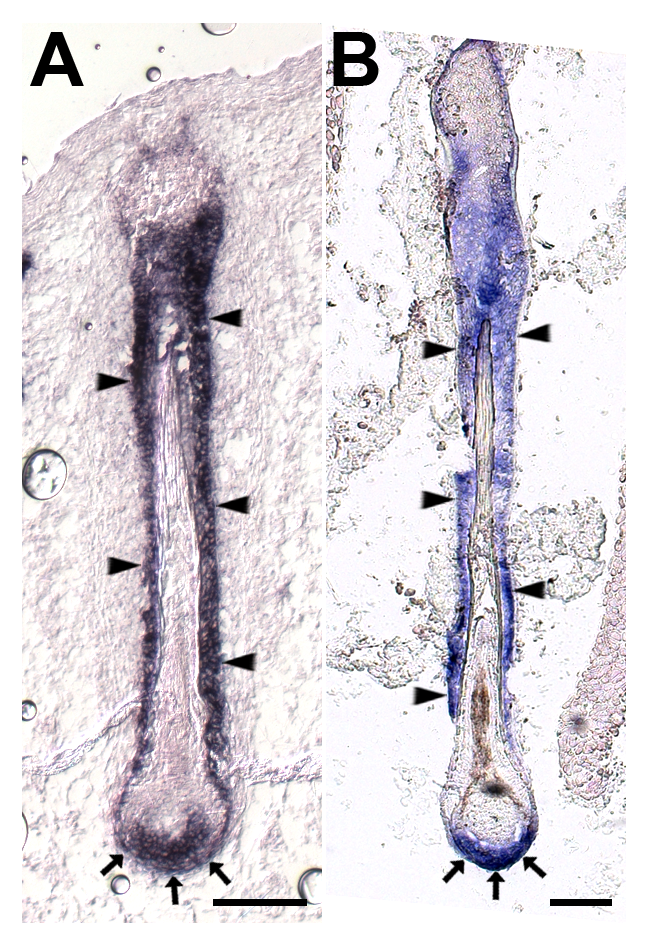

Supplement: Figure S1 — Lhx2 expression in whisker HFs mimics the expression pattern in pelage HF. (A) In situ hybridization analysis reveals Lhx2 expression in the ORS (arrow heads) and in matrix cells in the proximal part of the hair bulb (arrows) during whisker HF morphogenesis. (B) In situ hybridization analysis reveals Lhx2 expression in the ORS (arrow heads) and in matrix cells in the proximal part of the hair bulb (arrows) in an adult whisker HF. Scale bar, 100 µm. (1.41 MB TIF) [file pgen.1000904.s001.tif]

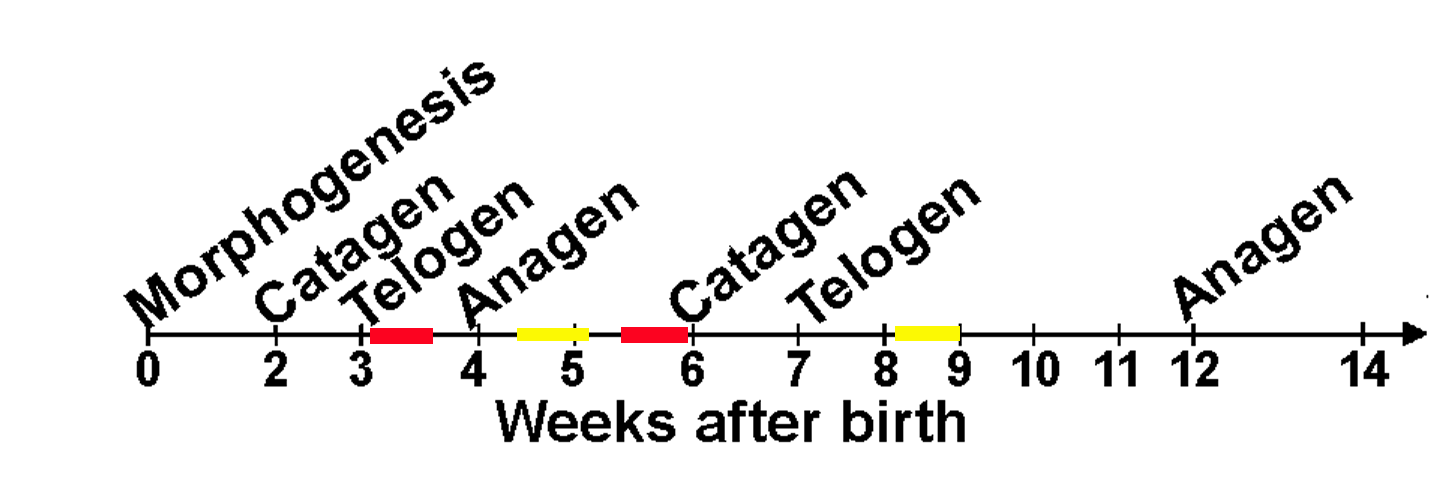

Supplement: Figure S2 — Time scale for the first postnatal and synchronized HF cycles in female C57BL/6 mice, and an overview of the strategy to conditionally inactivate the Lhx2 gene or induce transgenic expression of Lhx2. The time scale to illustrate the temporal progression through the postnatal HF cycles is adapted from [29]. The Lhx2 gene was conditionally inactivated by treating shaved back skin on CreER:Lhx2flox/flox and CreER:Lhx2flox/- mice with Tx during the first postnatal telogen phase in approximately 3-week-old mice (indicated by the first red bar between week 3 and 4). The progression of the first postnatal anagen phase was analysed in these mice and control Lhx2flox/flox and Lhx2flox/- animals at 5.5 to 6 weeks of age (indicated by the second red bar between week 5 and 6). To induce transgenic expression of Lhx2 in postnatal HFs the CreER:Z/Lhx2-GFP double transgenic mice were shaved on the back skin and treated with Tx in approximately 5 weeks old mice (indicated by the first yellow bar at 5 weeks). The effect of transgenic Lhx2 expression was analysed and compared to control single transgenic mice (CreER or Z/Lhx2-GFP) during the extended telogen phase at 8–9 weeks of age (indicated by the second yellow bar between week 8 and 9). (0.10 MB TIF) [file pgen.1000904.s002.tif]

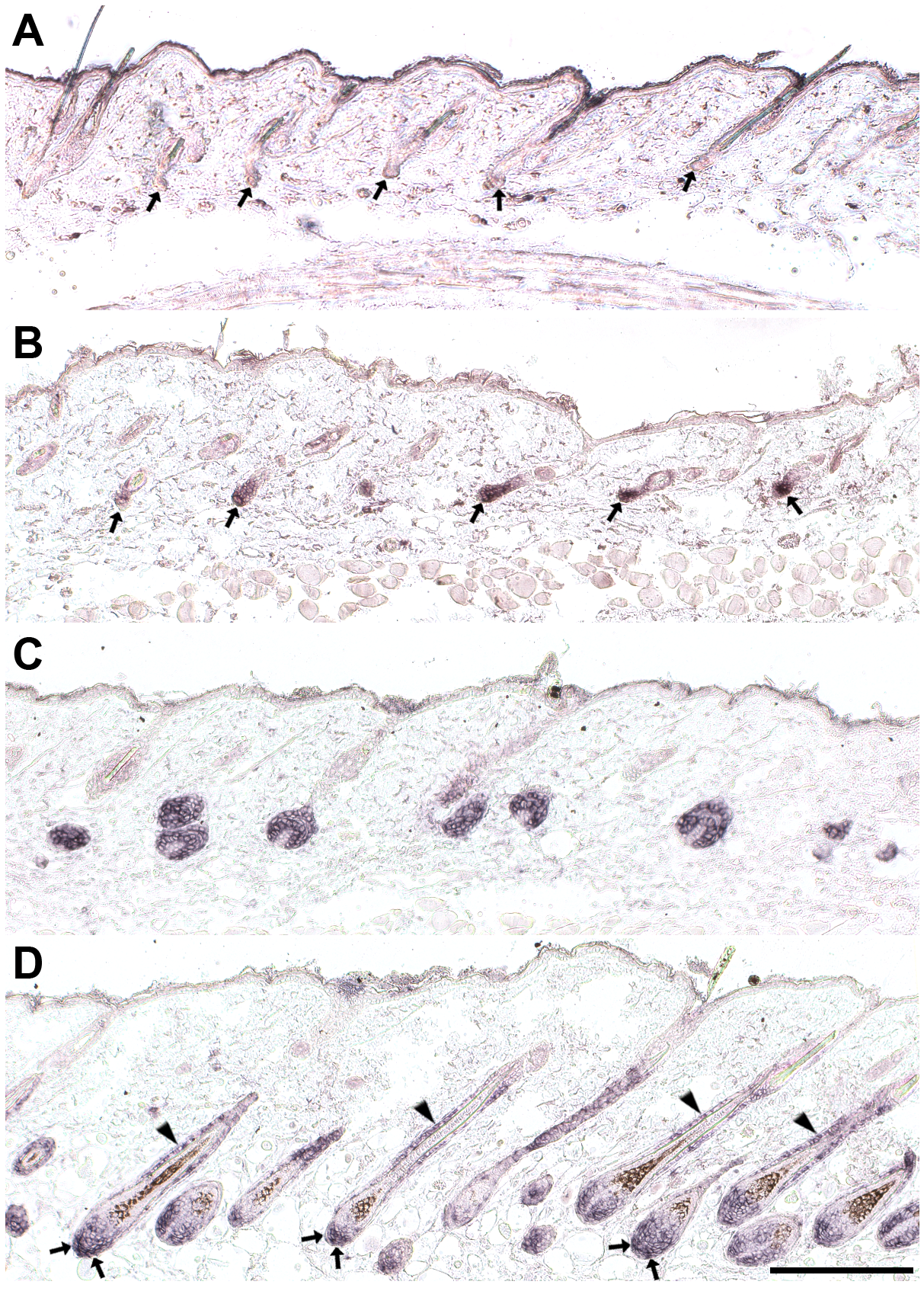

Supplement: Figure S3 — Over-view of Lhx2 expression pattern of HFs in early telogen, late telogen and early anagen Sub-stages I/II and III. (A) Analysis of Lhx2 expression in HFs in early telogen (3-week- and 1-day-old mice). Most mice at this age have their HFs in telogen (Table S1). No expression can be detected in any HF at this stage. (B) Analysis of Lhx2 expression in HFs in telogen in late telogen (3 week and 4 days old mice). Numerous mice have initiated anagen at this age (Table S1). Homogenous and distinct expression is detected in the secondary HG of all HFs at this stage (arrows). Shh is not expressed in HFs at this stage confirming that HFs are in telogen (Figure 2I). (C) Analysis of Lhx2 expression in HFs in anagen Sub-stages I-II (prior to pigment deposition). Lhx2 expression is detected in all HFs in the secondary HG and most epithelial cells in the down-growing part of the HF. Shh is expressed in all HFs at this stage (Figure 2M). (D) Analysis of Lhx2 expression in HFs in anagen Sub-stage III (all HFs contain pigment and most hair shaft has not reached the hair canal). Lhx2 expression is detected in cells in the ORS (arrow heads) and the proximal part of the hair bulb (arrows). Scale bar, 100 µm. (4.19 MB TIF) [file pgen.1000904.s003.tif]

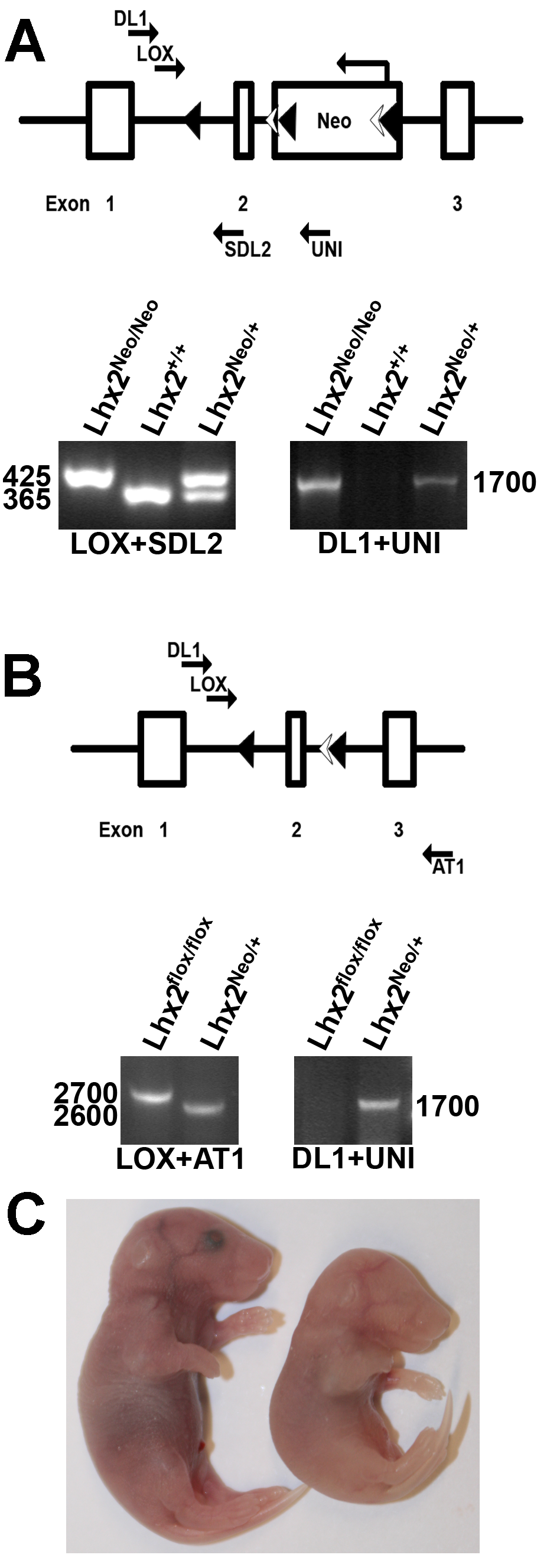

Supplement: Figure S4 — Description of the mouse strains with a hypomorphic allele of Lhx2 (Lhx2Neo), and a floxed allele of Lhx2 (Lhx2flox). (A) Description of the hypomorphic allele of Lhx2 locus containing the Neo gene between exon 2 and 3 in the opposite transcriptional orientation (upper panel). The location of the primers used to identify the WT allele and the Lhx2Neo allele are indicated (upper panel) and the PCR results to identify mice that are WT, heterozygous or homozygous for the Lhx2Neo allele are shown (lower panel). lox sites are indicated by black triangles and Flp sites are indicated by white arrow heads. (B) Description of the “floxed” Lhx2 allele (Lhx2flox) after Flp-mediated deletion of the Neo gene (upper panel). The location of the primers used to identify the Lhx2flox allele are indicated (upper panel) and the PCR results to distinguish mice that have the Lhx2Neo allele or the Lhx2flox allele are shown (lower panel). (C) Comparison between an E18.5 control embryo (left) and an Lhx2Neo/Neo embryo (right). The Lhx2Neo/Neo embryos develop the same eyeless phenotype as the Lhx2−/− embryos confirming that Lhx2 expression is significantly decreased. However, the anemia in Lhx2Neo/Neo embryos is less severe compared to the Lhx2−/− embryos and the expected number of live embryos can therefore be obtained at E18.5 when pelage HF morphogenesis is well established. (0.88 MB TIF) [file pgen.1000904.s004.tif]

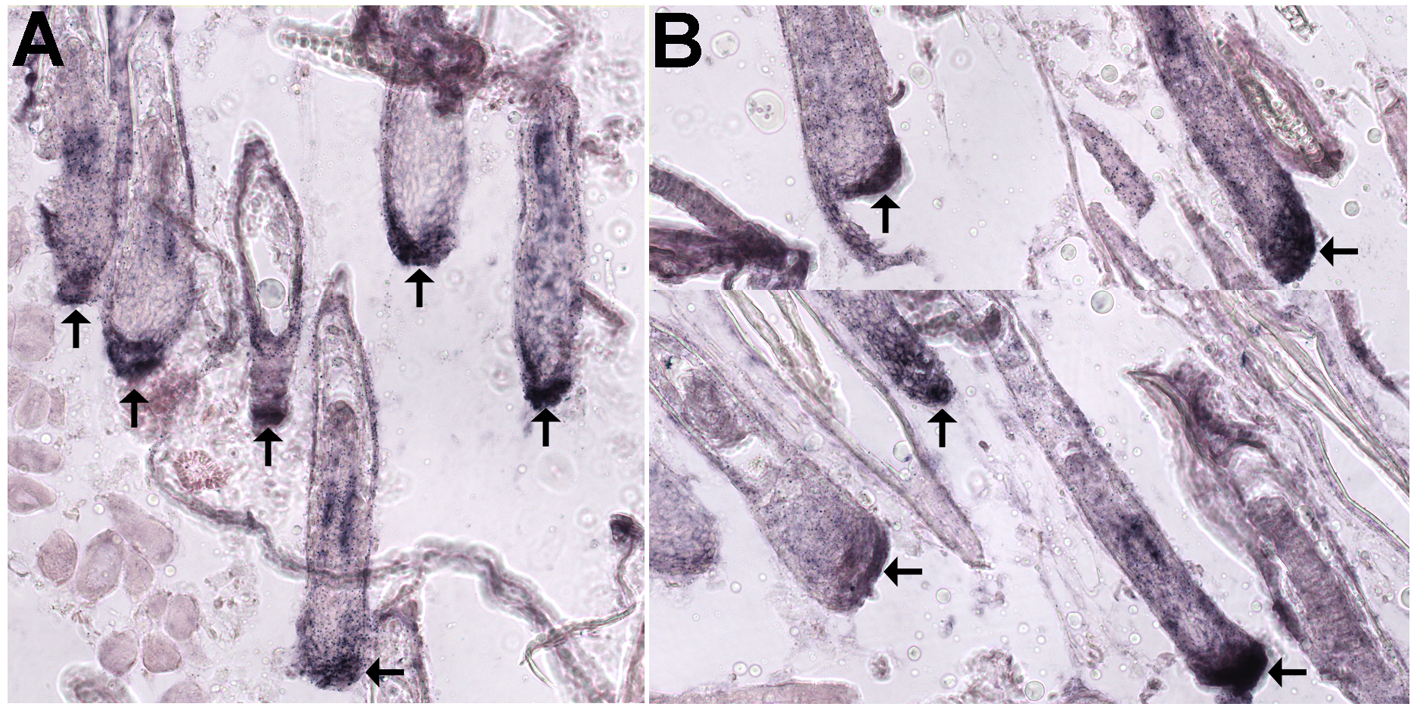

Supplement: Figure S5 — Conditional inactivation of Lhx2 is incomplete in Tx-treated CreER:Lhx2flox/flox mice leading to re-growth of some hair. In situ hybridization analyses on HFs from the shaved and Tx-treated area on CreER:Lhx2flox/flox mouse where hair started to re-grow. (A) In situ hybridization using the full length Lhx2 probe that detects both the WT allele and the mutant allele. (B) In situ hybridization using the probe restricted to exon 2 that only detects the WT allele (arrows), revealing incomplete inactivation of the Lhx2 gene leading to rescue of hair growth. (2.53 MB TIF) [file pgen.1000904.s005.tif]

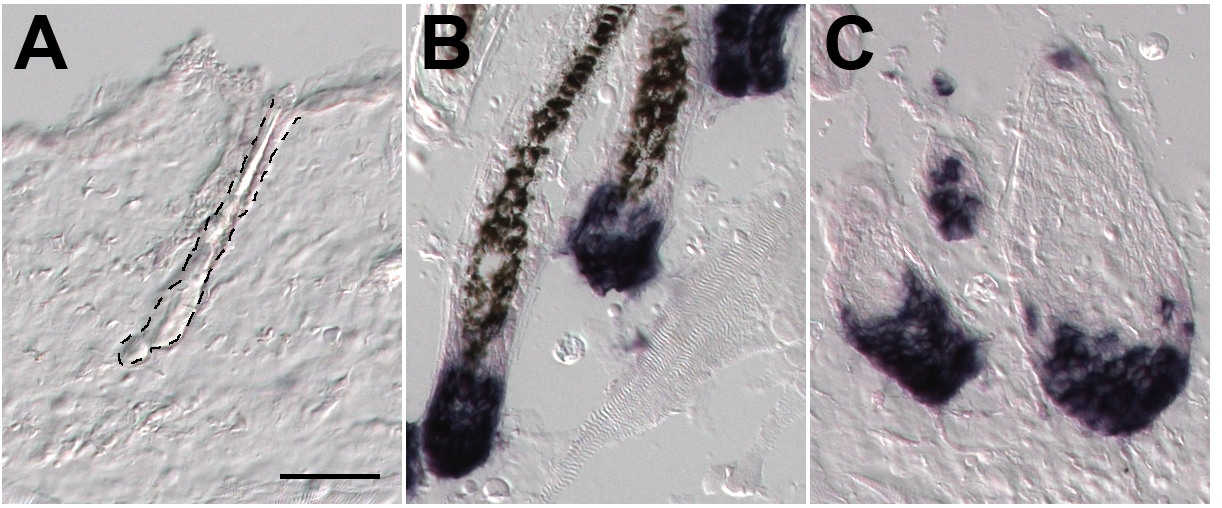

Supplement: Figure S6 — The S-phase-specific histone gene Hist1h3c is only expressed during anagen. In situ hybridization analyses of Hist1h3c expression of HFs in telogen (A) and anagen (B). (C) Hist1h3c is expressed in anagen HFs where Lhx2 has been conditionally inactivated in a similar pattern as in control HFs. (1.20 MB TIF) [file pgen.1000904.s006.tif]

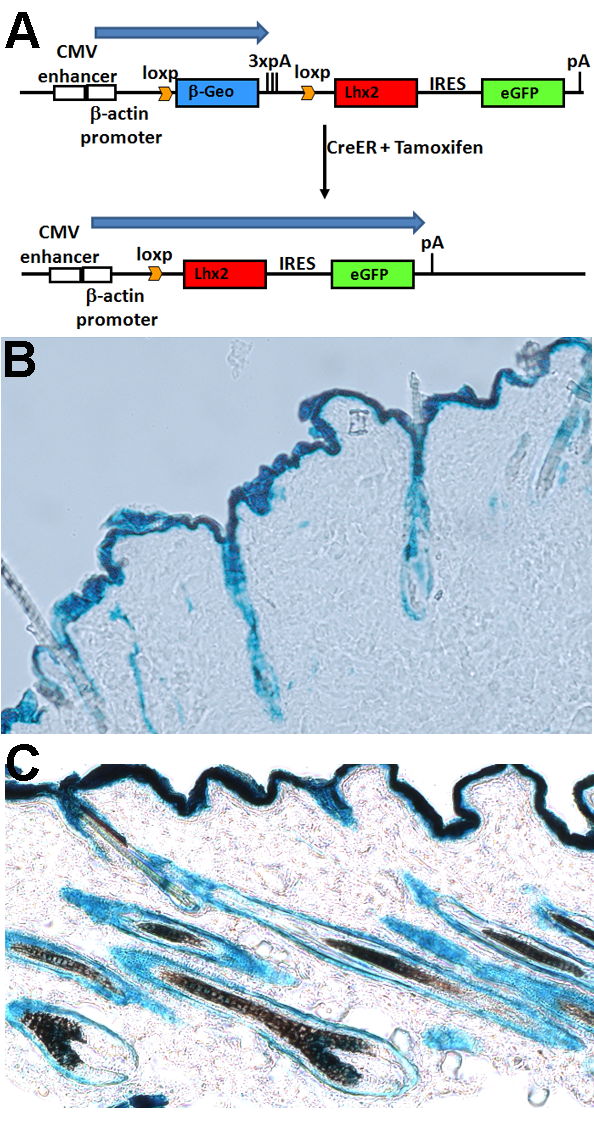

Supplement: Figure S7 — Generation of a transgenic mouse strain where Lhx2 expression can be induced in postnatal HF epidermis. (A) Schematic representation of the vector used to generate the Z/Lhx2-GFP transgenic mouse strain (upper panel) and the organisation of this vector after Cre-mediated recombination (lower panel). The blue arrows correspond to the mRNA that is generated before and after Cre-mediated recombination of this vector. The expression system is based on the Z/AP double reporter vector developed by Lobe and co-workers [39], where a floxed allele of β-Geo (encoding a β-galactosidase-Neomycin fusion protein) is followed by an expression cassette consisting of the Lhx2 cDNA, an internal ribosomal entry site (IRES) and green fluorescent protein (GFP) cDNA. Thus, DNA recombination by the Cre recombinase will delete the β-Geo gene and place Lhx2-GFP immediately downstream of the promoter/enhancer leading to its transcription. We generated a mouse strain transgenic for this vector (Z/Lhx2-GFP) that showed β-Gal activity in a variety of tissues including epidermis and in the epithelial portion of HFs in telogen (B) as well as anagen (C). The Z/Lhx2-GFP mouse strain was crossed with the CreER transgenic mouse strain where Tx-treatment of skin of the Z/Lhx2-GFP:CreER double transgenic mice will lead to activation of Lhx2 expression in HFs. (1.18 MB TIF) [file pgen.1000904.s007.tif]
