# Supplementary material for: Cyclic Expression of Lhx2 Regulates Hair Formation
Source: PLoS Genet. 2010 Apr 8;6(4):e1000904. doi: 10.1371/journal.pgen.1000904 (PMC2851574; doi:10.1371/journal.pgen.1000904)
Supplement: Table S1 — Hair cycle stage of back skin HFs in control mice at different ages. (0.10 MB PDF) [file pgen.1000904.s008.pdf]

Table S1. Hair cycle stage of back skin HFs in control mice at different ages.

**Mouse identity**	**Sex**	**Genotype**	**Age**	**Hair cycle phase Actual[1]**	**Hair cycle phase Predicted[2]**
1595-1	ND	*Nes-Cre*	1w. 1d.	Morphogenesis	Morphogenesis
1595-3	ND	*Z/Lhx2-GFP*	1w. 1d.	Morphogenesis	Morphogenesis
1595-4	ND	*Nes-Cre*	1w. 1d.	Morphogenesis	Morphogenesis
1595-5	ND	*WT*	1w. 1d.	Morphogenesis	Morphogenesis
1662-1	ND	*Nestin-Cre*	1w. 1d.	Morphogenesis	Morphogenesis
1662-2	ND	*Z/Lhx2-GFP*	1w. 1d.	Morphogenesis	Morphogenesis
1662-3	ND	*WT*	1w. 1d.	Morphogenesis	Morphogenesis
1662-4	ND	*WT*	1w. 1d.	Morphogenesis	Morphogenesis
3056-1	♀	*WT*	3w. 1d.	Telogen	Telogen
3056-2	♀	*WT*	3w. 1d.	Telogen	Telogen
3049-1	♂	*WT*	3w. 4d.	Anagen (I-II)	Telogen
3049-2	♂	*WT*	3w. 4d.	Anagen (III)	Telogen
3049-3	♀	*WT*	3w. 5d.	Telogen	Telogen
3049-4	♂	*WT*	3w. 5d.	Anagen (IV-V)	Telogen
3049-5	♂	*WT*	3w. 6d.	Anagen (IV-V)	Telogen
3347	♂	*Lhx2*$^{+/-}$	4w.	Anagen (IV-V)	Anagen
3346	♂	*Lhx2*$^{+/-}$	4w.	Anagen (VI)	Anagen
3348	♂	*Lhx2*$^{+/-}$	4w.	Anagen (VI)	Anagen
2694	♂	*Z/Lhx2-GFP*	4w. 1d.	Telogen	Anagen
2664	♂	*WT*	4w. 3d.	Anagen (VI)	Anagen
B3	♀	*Lhx2*$^{flox/flox}$	4w. 3d.	Anagen (I-III)	Anagen
2691	♂	*WT*	5w.	Anagen (VI)	Anagen
B1	♂	*Lhx2*$^{flox/-}$	5w.	Anagen (III)	Anagen
B2	♀	*Lhx2*$^{flox/-}$	5w. 1d.	Anagen (V-VI)	Anagen
1858	♂	*Nes-Cre*	5w. 3d.	Anagen (VI)	Anagen
2687	♀	*WT*	5w. 3d.	Anagen (VI)	Anagen
B10	♀	*Lhx2*$^{flox/-}$	5w. 3d.	Anagen (VI)	Anagen
1859	♂	*WT*	5w. 3d.	Catagen (III-IV)	Anagen
1973	♀	*WT*	5w. 5d.	Anagen (VI)	Anagen
1971	♂	*WT*	5w. 5d	Catagen (V-VI)	Anagen
S2	♂	*Lhx2*$^{flox/-}$	5w. 6d.	Anagen (VI)	Anagen
S3	♀	*Lhx2*$^{flox/flox}$	5w. 6d.	Anagen (VI)	Anagen
2689	♀	*WT*	6w.	Telogen	Catagen
2839	♂	*CreER*	6w. 4d.	Catagen (II-III)	Catagen
3087	♀	*WT*	6w. 6d.	Anagen (VI)	Catagen
3088	♀	*WT*	6w. 6d.	Anagen (VI)	Catagen
3086	♂	*WT*	6w. 6d.	Telogen	Catagen
2945	♂	*Z/Lhx2-GFP*	7w.	Anagen (VI)	Telogen
2954	♀	*WT*	7w.	Anagen (VI)	Telogen
2955	♀	*Z/Lhx2-GFP*	7w.	Anagen (VI)	Telogen

2956	♀	*CreER*	7w.	Anagen (VI)	Telogen
2946	♀	*CreER*	7w.	Telogen	Telogen
2950	♂	*WT*	7w.	Telogen	Telogen
2951	♂	*CreER*	7w.	Telogen	Telogen
2803	♂	*CreER*	7w. 3d.	Telogen	Telogen
2804	♂	*CreER*	7w. 3d.	Telogen	Telogen
2308	♂	*Lhx2*$^{+/-}$	8w. 1d.	Telogen	Telogen
1796	♂	*Z/Lhx2-GFP*	8w. 2d.	Telogen	Telogen
3149	♀	*Z/Lhx2-GFP*	8w. 2d.	Telogen	Telogen
3151	♀	*WT*	8w. 2d.	Telogen	Telogen
3152	♀	*CreER*	8w. 2d.	Telogen	Telogen
1434	♂	*WT*	8w. 4d.	Telogen	Telogen
1435	♂	*CreER*	8w. 4d.	Telogen	Telogen
2	♂	*Z/Lhx2-GFP*	9w.	Telogen	Telogen
3	♂	*WT*	9w.	Telogen	Telogen
5	♀	*CreER*	9w.	Telogen	Telogen
6	♀	*Z/Lhx2-GFP*	9w.	Telogen	Telogen
7	♀	*WT*	9w.	Telogen	Telogen
2699	♀	*CreER*	9w.	Anagen (VI)	Telogen
2701	♀	*CreER*	9w.	Telogen	Telogen
2786	♂	*CreER*	9w.	Telogen	Telogen
2845	♂	*WT*	9w.	Telogen	Telogen
2848	♀	*CreER*	9w.	Telogen	Telogen
2849	♀	*CreER*	9w.	Telogen	Telogen
3387	♂	*WT*	9w.	Telogen	Telogen
3392	♂	*Z/Lhx2*	9w.	Telogen	Telogen
3444	♂	*Z/Lhx2*	9w.	Telogen	Telogen
3449	♀	*CreER*	9w.	Telogen	Telogen
3453	♀	*Z/Lhx2*	9w.	Telogen	Telogen
3487	♂	*Z/Lhx2*	9w.	Telogen	Telogen
3489	♂	*CreER*	9w.	Telogen	Telogen
3490	♂	*Z/Lhx2*	9w.	Telogen	Telogen
3488	♂	*CreER*	9w.	Telogen	Telogen
3491	♂	*WT*	9w.	Telogen	Telogen
1419	♂	*WT*	9w. 1d.	Telogen	Telogen
1420	♂	*Z/Lhx2-GFP*	9w. 1d.	Telogen	Telogen
1787	♂	*Nestin-Cre*	9w. 1d.	Telogen	Telogen
2805	♀	*CreER*	9w. 1d.	Telogen	Telogen
3155	♂	*Lhx2*$^{+/-}$	9w. 2d.	Telogen	Telogen
3173	♀	*Lhx2*$^{+/-}$	9w. 6d.	Telogen	Telogen
3217	♂	*WT*	10w. 1d.	Telogen	Telogen
3148	♂	*WT*	10w. 4d.	Telogen	Telogen
2787	♀	*CreER*	10w. 5d.	Telogen	Telogen
1923	♀	*Nes-Cre*	11w.	Telogen	Telogen
3218	♂	*WT*	11w.	Telogen	Telogen
1958	♀	*Nes-Cre*	11w. 6d.	Anagen (IV-V)/Telogen	Telogen
3132	♂	*WT*	12w. 2d.	Telogen	Anagen

1852	♂	*Z/Lhx2-GFP*	12w. 4d.	Telogen	Anagen
1856	♀	*WT*	12w. 4d.	Telogen	Anagen
1968	♀	*Z/Lhx2-GFP*	13w.	Telogen	Anagen
1751	♂	*Z/Lhx2-GFP*	13w. 5d.	Telogen	Anagen
1646	♂	*Z/Lhx2-GFP*	14w. 4d.	Telogen	NA
1965	♂	*Z/Lhx2-GFP*	14w. 6d.	Telogen	NA
1967	♂	*Z/Lhx2-GFP*	15w. 6d.	Anagen (IV-V)/Telogen	NA
1790	♀	*Nes-CRE*	16w. 2d.	Anagen (VI)	NA
1857	♀	*Nes-Cre*	16w. 2d.	Anagen (VI)	NA
1792	♀	*Nes-Cre*	18w.	Telogen	NA
1590	♀	*Z/Lhx2-GFP*	18w. 4d.	Telogen	NA
1644	♂	*Z/Lhx2-GFP*	20w. 1d.	Telogen	NA
1586	♂	*Nes-Cre*	26w. 3d.	Telogen	NA

[1] Hair cycle stage and sub-stage is determined according to Müller-Röver et al. [29]. Both an anterior and a posterior part of back skin were analysed except in the 1w. 1d. old mice where a midsection of the back skin was analysed.

[2] Predicted hair follicle cycle in female C57BL/6 mice according to Müller-Röver et al. [29].

ND Not determined

NA Not applicable, hair follicle cycles becomes increasingly asynchronous at this age
